# Digital Twin Approach for Operation and Maintenance of Transportation System—Systematic Review

**DOI:** 10.3390/s24186069

**Published:** 2024-09-19

**Authors:** Sylwia Werbińska-Wojciechowska, Robert Giel, Klaudia Winiarska

**Affiliations:** Faculty of Mechanical Engineering, Wroclaw University of Science and Technology, Wyspianskiego 27, 50-370 Wroclaw, Poland; robert.giel@pwr.edu.pl (R.G.); klaudia.winiarska@pwr.edu.pl (K.W.)

**Keywords:** digital twin, operation and maintenance, transportation system, systematic review analysis, air transportation, railway transportation, land transportation, in-house logistics, water and intermodal transportation, supply chains operation, PRISMA guidelines

## Abstract

There is a growing need to implement modern technologies, such as digital twinning, to improve the efficiency of transport fleet maintenance processes and maintain company operational capacity at the required level. A comprehensive review of the existing literature is conducted to address this, offering an up-to-date analysis of relevant content in this field. The methodology employed is a systematic literature review using the Primo multi-search tool, adhering to the Preferred Reporting Items for Systematic Reviews and Meta-Analyses (PRISMA) guidelines. The selection criteria focused on English studies published between 2012 and 2024, resulting in 201 highly relevant papers. These papers were categorized into seven groups: (a) air transportation, (b) railway transportation, (c) land transportation (road), (d) in-house logistics, (e) water and intermodal transportation, (f) supply chain operation, and (g) other applications. A notable strength of this study is its use of diverse scientific databases facilitated by the multi-search tool. Additionally, a bibliometric analysis was performed, revealing the evolution of DT applications over the past decade and identifying key areas such as predictive maintenance, condition monitoring, and decision-making processes. This study highlights the varied levels of adoption across different transport sectors and underscores promising areas for future development, particularly in underrepresented domains like supply chains and water transport. Additionally, this paper identifies significant research gaps, including integration challenges, real-time data processing, and standardization needs. Future research directions are proposed, focusing on enhancing predictive diagnostics, automating maintenance processes, and optimizing inventory management. This study also outlines a framework for DT in transportation systems, detailing key components and functionalities essential for effective maintenance management. The findings provide a roadmap for future innovations and improvements in DT applications within the transportation industry. This study ends with conclusions and future research directions.

## 1. Introduction

The dynamics of the transportation market and the growing demands of customers pose significant challenges for transportation companies in the context of maintaining the durability and reliability of transport means. The transportation industry is subject to continuous changes resulting from various factors, such as technological advancements, changing regulations, and consumer trends. In recent years, the dynamic development of the transportation sector can be observed as driven by ongoing globalization, the growth of international trade, and increasing societal mobility. Transportation companies face intensified competition from both traditional and new, innovative entities. Market dynamics force them to constantly adapt to changing conditions and seek new solutions and technologies to maintain a competitive position [1].

Simultaneously, the growing demands of customers on transportation companies are becoming increasingly diverse. Customers expect quick and timely delivery of goods and high-quality service, safety, and flexibility in adapting services to individual needs. High customer demands require transportation companies to ensure the efficient operation of their fleets and the quality of services provided at every stage of the transportation process. This entails implementing fast and efficient customer service procedures and having a modern vehicle fleet with effective maintenance management and quick emergency response. In this context, ensuring the high maintainability and reliability of the transportation fleet becomes a key issue [2,3,4].

The challenges related to maintaining high maintainability and reliability of the transportation fleet are significant for companies operating in the transportation sector due to the dynamic nature of the transportation environment and the variety of factors affecting vehicle performance. One of the main issues associated with maintaining the transportation fleet’s reliability is the vehicle fleet’s aging. Transport vehicles are operated under various weather and road conditions, leading to natural wear and degradation of mechanical parts and electronic components. Over time, the risk of failures and downtimes increases, negatively impacting the operational efficiency of transportation companies [5].

Another significant issue is the complexity of maintenance processes for the vehicle fleet. Regular technical inspections and repairs are required to ensure operational readiness and the necessary level of vehicle safety. Managing these maintenance processes is often demanding and time-consuming, especially when providing the proper maintenance level for large transportation fleets operating on diverse routes and under various operational conditions [6].

Additionally, the necessity for a quick response in case of failures and unforeseen situations is also problematic. Vehicle downtimes can lead to delivery delays, generating costs and negatively impacting the company’s reputation. Therefore, transportation companies must take appropriate measures to minimize the risk of failures and downtimes and ensure the operational continuity of their fleets. Implementing modern methods and technologies, such as digital twins (DTs), can improve the efficiency of fleet maintenance processes and maintain the company’s operational capabilities at the required level. Investments in modern technological solutions allow for improved fleet durability and reliability, minimized operational costs, and increased market competitiveness.

Recently, numerous studies and publications have emerged in the transportation sector, focusing on maintenance management and modeling to enhance the efficiency of maintenance processes (for a comprehensive review, see, for example, refs. [6,7]). The search for English language review publications in the Scopus database based on searching the following keywords—“*transportation OR transport*” AND “*maintenance OR maintenance management OR condition monitoring OR predictive maintenance*” AND “*review OR state of art OR current state*”—allowed for 24 relevant records to be identified. The identified papers were published from 2010 to 2024. The content analysis of these reviews revealed that most of these reviews are focused on specific transport sectors—railway maintenance [8,9,10,11,12,13,14], air transportation [15], road transportation [16,17,18], electric vehicles and fuel cell condition monitoring [19,20], and water and intermodal transport [21,22]. In addition, a few reviews are focused on transportation infrastructure maintenance [23,24,25,26,27,28,29,30]. However, there is a notable absence of comprehensive review articles addressing the application of digital twins in transportation system operation and maintenance especially in the context of in-house logistics systems. Despite the growing interest in digital twin technology and its potential benefits for the transportation sector, the current literature lacks thorough reviews that summarize existing knowledge and identify research gaps in this specific area. Only two of the identified reviews focus on the aspect of DT use in the maintenance of transportation systems. First, the authors in [3] concentrate on the integration of Digital Twins and their impact on the evolution of transportation asset management systems. Secondly, Selvam et al. [31] provide an in-depth analysis of the current advancements in incorporating digital twins into the maintenance of integrated chargers in electric vehicles. 

This study provides a comprehensive overview of academic research on the application of digital twins in the operation and maintenance of transportation systems, with special emphasis on internal transportation. The primary goal is to identify key research trends in this field and suggest potential future research directions. Additionally, based on the literature review, a framework for digital twins in the internal transport sector is developed, drawing from the physical asset management concept and ISO/DIS 23247 standards [32,33,34,35]. Consequently, this paper contributes to the existing knowledge on digital twins in transportation systems in three ways: (1) identifying the major research trends related to DT applications in the operation and maintenance of transportation systems, with a focus on in-house logistics; (2) outlining future research directions for the study of DT in transportation systems operation and maintenance; and (3) developing a framework for DT in transportation system maintenance management.

Based on these objectives, the research questions are as follows:

RQ1: What is the state of the literature on digital twin use in transportation systems operation and maintenance between 2012 and 2024?

RQ2: What are the main research and knowledge gaps in DT use in transportation systems operation and maintenance, especially in the context of in-house logistics? 

RQ3: Which aspects of DT modeling require further advancement to address future challenges in transportation systems?

RQ4: What scope should the framework for digital twin for maintenance management of transportation systems have?

This paper addresses the research questions posed above by employing bibliometric performance analysis and systematic analysis using the PRISMA method (Preferred Reporting Items for Systematic Reviews and Meta-Analyses) [36]. This approach is designed to summarize and pinpoint the key research areas within the identified application fields.

In summary, the article is structured into seven sections. Following the Introduction (Section 1), the Theoretical Background (Section 2) outlines the concept of digital twins and explores their application across various transportation sectors. The Review Methodology (Section 3) details the primary methods used for the review, including the strategy for the literature search and the criteria used to assess the relevance of the analyzed documents. Section 4 presents the main findings of the systematic literature review for the selected papers within the seven identified application fields. Section 5 then discusses the results related to these application fields, identifying gaps in the literature and knowledge. Section 6 introduces a newly developed DT framework for the maintenance of transportation systems. The final section, Conclusions (Section 7), provides a summary of contributions, outlines limitations, and offers recommendations for future research.

## 2. Theory Background

### 2.1. Digital Twin Concept—Introduction 

Digital twin (DT) is one of the key Industry 4.0 technologies. Although DT has gained a lot of interest in many sectors in the last five years, the first proposals for the concept were made in 1991. The history of the origin and evolution of technology to its current form can be read in [37,38,39,40,41]. 

The DT concept is relatively new; no coherent definition has yet been created. At the same time, it is constantly evolving due to technological advances, industry needs, and user needs, so the possibilities for its use have changed over the past few years [40]. The definitions presented in the literature and the essence of the concept are often related to the area in which it is applied [42]. Among others, examples of implementing the DT concept in practice can be found in [42,43,44,45,46]. Most publications on the areas of DT implementation are related to production systems. However, the concept is also increasingly appearing in agriculture and medicine. In addition, DT also finds applications in psychology [47]. 

In this paper, the authors focused on transport and logistics issues. In this context, the definitions often used in the literature target the specific transport branches for which the DT solution is developed. Therefore, it can be seen that some of the definitions tend to contradict each other. Hence, based on a thorough analysis of the definitions provided by D. Jones [46] for this work, it is assumed that a digital twin (DT) is a virtual representation of the actual process/asset/system based on Industry 4.0 technology use, where such activities as data sharing, simulation, time-based monitoring, data analysis, testing, and optimization are included. The combination of these technologies and activities aims at real-time monitoring, control, prediction, optimization, and more informed and faster operational decision-making [48].

**Figure 1:**
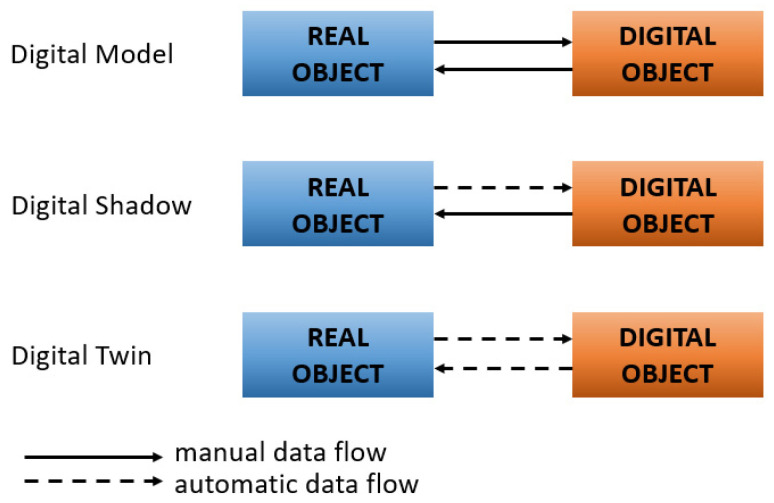
Data flow in different modes of integration. Source: own contribution based on [38].

The digital twin of any object in its basic version is built from three basic elements: a physical object (resource/asset), a virtual representation of the object (model), and the connection between them [43]. The represented object can be a product, process, or system. A DT is a virtual representation of any real object that encompasses all its features [37,49,50]. In addition, data are automatically shared in real time between the physical object and the digital counterpart [38,40]. This factor distinguishes a digital twin from a digital model (DM) or digital shadow (DS). This means that a DT is dynamic. In a DM, the interaction between the real object and its digital copy is manual. It is merely a virtual representation of the real object. In addition, the DM does not process input data and cannot react to changes in the real object [51]. In the case of a DS, the data transfer from the real object to the digital object is automatic, while the feedback is already manual. This allows for an accurate virtual representation of the real processes; however, the feedback is not provided automatically [51]. Figure 1 graphically illustrates the data flow in the concepts discussed. In the literature, the DM is seen as part of the DT, which enables the virtual visualization of the real object, and the DS is referred to as a digital design aiming at a DT, but it does not meet all the assumptions of a DT [51]. 

The improvement of the DT concept and the success of its implementation is made possible by the rapid development of other Industry 4.0 technologies on which DT is based, i.e., IoT, big data, or machine learning [52,53]. The data that DT uses comes from the real-world object, the model, and information about historical operations. Data from the real-world object are obtained using various sensors that provide real-time information. Additionally, through the use of IoT, which enables data collection and transmission, as well as data sharing, DT can collect information about different objects and their models [54]. The real-time transmission of data allows for the DT to be updated in real-time so that it does not deviate from the real object and accurately reflects its performance level or, e.g., degradation level [37]. By constantly transmitting a large quantity of new data from different sources, it is possible to maintain the dynamic nature of the DT. This provides the possibility to monitor the object and to accurately reflect it digitally in real-time. With the diversity of data sources, access to big data is necessary. All the collected data are analyzed through machine learning, and a dynamically changing virtual model is created based on this. The next step is to generate conditions that have not yet occurred in reality. The behavior of the object operating under the given conditions is also studied. In addition, problems that may arise under the given operating conditions are predicted [37,49]. This makes it possible to detect and eliminate errors in the virtual system before they occur in the real process [37,55]. Finally, some solutions are proposed to minimize the possibility of failure occurrence and completely prevent the identified problems [37]. Thanks to the bidirectional automatic connection between the physical and the digital object, information from simulations and predictions is transferred to the physical object, which is used in the operational process [56]. 

**Figure 2:**
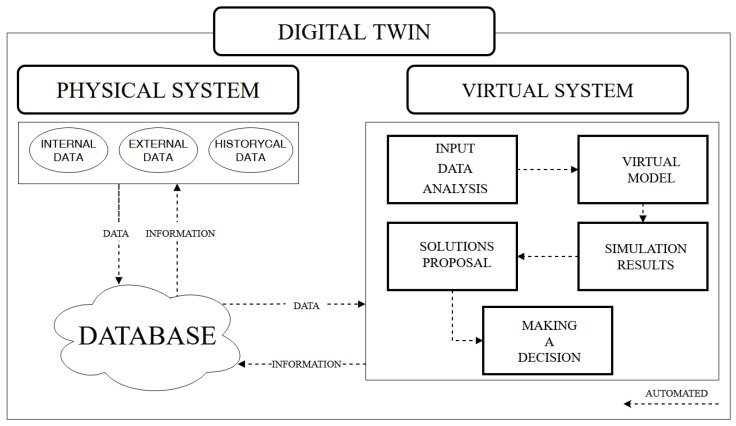
The digital twin operation concept. Source: own contribution based on [51,59].

The processes discussed above are the basic scope of DT functionality. These activities can also take place using machine learning algorithms [57]. Figure 2 illustrates the concept of DT operation. In addition, the accuracy and usability of DT are highly dependent on the quality of the sensor data provided by the real object [58]. Poor accuracy of this data or sensor failures cause interference with real-time monitoring of the object. Additionally, they can even lead to the failure of the physical object as it is operated based on feedback from the DT [56].

At the same time, an important aspect in the context of DT definition and development is the reference to the product lifecycle. Identifying the phases of DT development in relation to the product lifecycle allows for a clear definition of the basic tasks of DT in an organization. In addition, a DT may accompany its real-life twin from the early stages of its development, so it is beneficial to distinguish the phases of DT development throughout the object’s lifecycle. 

**Figure 3:**
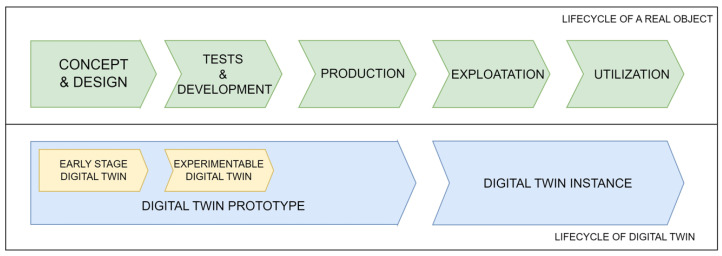
Digital twin lifecycle in relation to the lifecycle of an object. Source: own contribution based on [60,61,62].

DT is applicable throughout the whole lifecycle of a physical object, from the design phase through the production and operation phases to its disposal [43]. In the publications [60,61,62], the authors presented different stages of DT maturity. Based on their research results, the DT lifecycle was developed, which coincides with the lifecycle of the real object (Figure 3).

The first phase of the DT lifecycle is the digital twin prototype (DTP). It is designed for the object design activities. At this phase, the real object does not yet exist in its physical form. It only exists as a concept and design in virtual space, such as DTP [60]. Based on the information from the DTP, a physical object is developed that duplicates the digital version. In [61,62], DT types that partly overlap with the DTP assumptions are proposed. However, due to their purpose, they can be said to be only part of DTP. Early-stage digital twin (ESDT) corresponds to a real object’s conception and design phase. It aims to generate information and evaluate proposed solutions and concepts. This is possible through early simulation and analysis [61]. Experimentable digital twin (EDT) corresponds to the research and development phase of the lifecycle of a real object. An EDT is a virtual prototype intended primarily to test and verify a designed object or system in its target operational environment [62]. 

The DT maturity stage corresponding to the exploitation and disposal phase of the real object is the digital twin instance (DTI). The DTI represents a concrete physical object and evolves throughout its lifetime until disposal. It contains past, current, and projected operational data and service records [60].

The concept of DT has been gaining particular interest over the last five years, whether in terms of the development of the approach itself, the building of customized architectural solutions, DT characteristics, design principles, or future challenges. As a result, a number of articles can be distinguished that aim to summarize the basic literature on DT designing, modeling, and implementation. For example, a comparison of different definitions describing DT is presented in [42], the architecture and modeling approach are described in [50,63,64,65], and the link between Industry 4.0 development and DT is described in [55,66]. In contrast, DT in Industry 5.0 is investigated in [54,67,68]. The main challenges are reviewed, e.g., in [49,69,70]. A summary of recent review papers that focus on DT concept definition, modeling, architecture, or research challenges is given in Table 1. 

**Table 1:** A summary of recent papers focused on providing an overview of the literature in the area of the digital twin concept.

Ref.	Publication Year	Research Objectives	Methodology Used	Databases Analyzed	Papers Analyzed
[42]	2019	Analysis of the state-of-the-art definitions of DT, investigation of the main characteristics of DT, and exploration of DT applications	Systematic literature review	Google Scholar	75
[53]	2020	Survey of the state-of-the-art of major definitions, specifications, and implementations of the DT concept in several technological areas with an attempt to consolidate the major features of the DT concept as it has emerged in different industries	Survey review	n/a	140
[52]	2021	Review of digital twinning, particularly focusing on the role of AI-ML and big data	Systematic literature review	IEEE Xplore, ACM digital library, Scopus (ScienceDirect, Elsevier), SpringerLink, Hindawi, IGI-Global, Taylor & Francis Online, Wiley online library, the US patents database	157
[71]	2021	Review the evolution of DTs in tomorrow’s digital factories and research toward implementing context-aware, autonomous, and adaptive DTs.	State-of-the-art review	n/a	n/a
[72]	2021	Review of DT history, definitions, models, types of key enabling technologies, and applications	State-of-the-art review	n/a	n/a
[73]	2021	Review of existing reviews relating to DT	Meta-review	AISeL, IEEE Xplorer, Science Direct, Springer Link	24
[49]	2022	Review of various DT features and current approaches, the shortcomings and reasons behind the delay in the implementation and adoption of digital twin and development of DT reference model	State-of-the-art review	n/a	n/a
[64]	2022	A comprehensive view of the DT technology and its implementation challenges and limits in the most relevant domains and applications in engineering and beyond	Systematic literature review	ResearchGate, MDPI, Science Direct, and ProQuest	84
[69]	2022	Review of the current state of digital twins, describing the terms digital model and digital shadow; review the concepts of Internet of Things (IoT) and Industry 4.0	Overview	n/a	n/a
[74]	2022	Survey of potential threats associated with the DT paradigm, taking into consideration its functionality layers and the operational requirements	Survey review	n/a	n/a
[75]	2022	Review of research and applications of DT in smart manufacturing	Bibliometric review	Web of Science Core Collection	3763/top 100
[76]	2022	Review of recent advancements in the DT in the context of technology, market potential and trends, applications, and case studies	Survey review	n/a	n/a
[77]	2022	Reviewing of SLRs on DTs and analyzing the body of related work with respect to the presented research scope	Meta-review	Google Scholar, Web of Science database	14
[70]	2023	Review of digital twin definition, emphasizing important characteristics; analysis of techniques, trends, and future research directions	Systematic review according to Kitchenham’s guidelines [78]	Google Scholar	31
[79]	2023	Literature review on Digital Twins in the context of intelligent automation use in different industries	Narrative literature review	Scopus database, Google Scholar	n/a
[80]	2023	Review on data management solutions proposed in the DT context	Systematic literature review	ACM Digital Library, IEEE Digital Library, Onepetro, Scopus, Science Direct, Web of Science database	61

### 2.2. Digital Twin Concept—Implementation Areas

**Figure 4:**
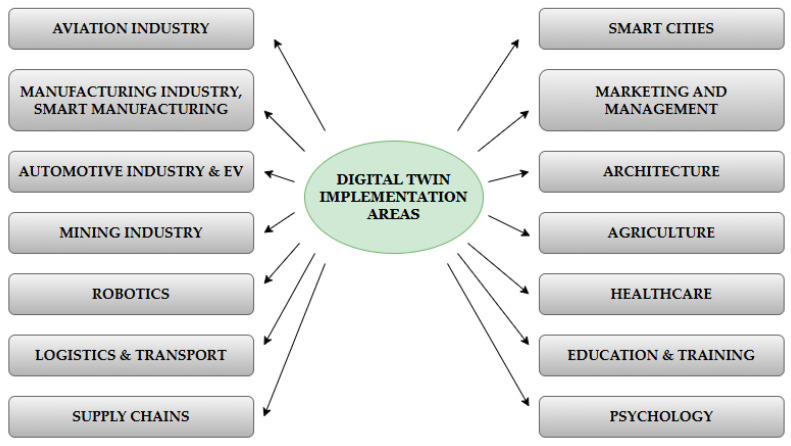
The digital twin concept’s main implementation areas. Source: own contribution.

As has already been mentioned, DT is currently being implemented in various industrial sectors due to its broad applicability. As an innovative approach, it provides new solutions to problems faced by numerous industries, ranging from designing new products or even factories to issues related to optimizing the operation of selected technical systems or organizations. Due to such a high level of interest in the possibilities of DT implementation, we can currently distinguish a number of works summarizing recent developments in this area (see, e.g., refs. [64,81,82,83,84,85]). At the same time, based on the literature analysis carried out, it was possible to propose basic areas of DT practical application (Figure 4).

**Figure 5:**
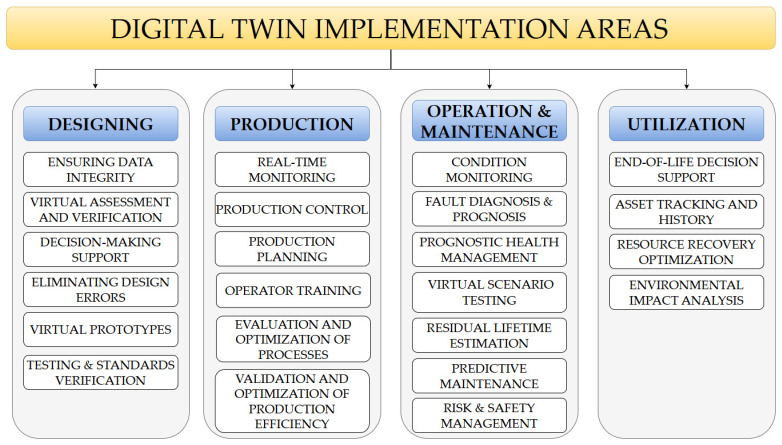
The digital twin concept’s main implementation areas in relation to the object’s lifecycle. Source: own contribution based on [43,117].

Digital twin technology, which creates detailed and dynamic virtual replicas of physical systems, is revolutionizing numerous industries by providing real-time insights and predictive capabilities. As shown in Figure 5, this technology is being implemented across a wide range of sectors, enhancing efficiency, performance, and innovation in diverse applications.

Digital twins are increasingly applied across various industries to enhance operations and efficiency. In aviation, they monitor aircraft conditions, predict maintenance needs, and optimize operations, improving safety and reliability [86,87,88]. In smart manufacturing, digital twins enable real-time insights, predictive maintenance, and workflow optimization, integrating IoT, AI, and machine learning for greater productivity and flexibility [44,89,90,91,92]. The automotive industry benefits from digital twins in vehicle design, manufacturing, and maintenance, supporting innovations in electric vehicles and autonomous driving [93,94,95,96,97]. In the mining industry, digital twins improve safety, resource management, and operational efficiency [98]. In logistics and transportation, they optimize routes, fleet management, and predictive maintenance [99,100,101]. Additionally, digital twins enhance supply chain management by providing real-time tracking and improving demand forecasting [99,100,102,103,104,105]. Other applications include robotics, where they are used for simulation, testing, and real-time control of robotic systems [106,107], smart cities [108], and in education, training, healthcare, and psychology, where they offer immersive learning experiences and personalized treatments (see, e.g., refs. [42,109,110,111,112,113,114,115,116]).

In addition, due to the introduction of the DT lifecycle given in Section 2.1, it is worth investigating how the implementation areas are aligned with the phases of the lifecycle of an object. DTs provide invaluable insights and optimizations throughout the object’s lifecycle—design, production, operation, and end-of-life. DTs allow for virtual prototyping and testing in the design phase, reducing time and costs. During production, they enable real-time monitoring and quality control, enhancing efficiency. DTs facilitate predictive maintenance and performance optimization in the operational phase, extending product lifespan. Finally, at the end-of-life phase, DTs assist in planning for recycling or disposal. This comprehensive integration across the product lifecycle highlights the transformative potential of digital twins. Figure 5 shows for which purposes DT was applied during the different phases of the object’s lifecycle. 

From the point of view of this article, which focuses on maintenance issues, the third phase of the object’s lifecycle is of most interest.

With increasing frequency, authors describe the possibility of using DT in maintenance operations and management. In the context of maintenance, DT plays an important role because it offers the possibility to evolve the way maintenance is carried out. This means the possibility of moving towards more advanced maintenance strategies such as predictive or prescriptive maintenance [118,119]. Preventive maintenance without the use of DT is only used as a calculation tool to analyze the condition of an object and predict faults [120]. The results then obtained do not reflect the long-term dynamically changing real-world data. DT provides more intelligent maintenance management than in the case of predictive maintenance implementation. This is due to the automatic analysis of the collected data related to the operation, technical condition, or facility utilization. Based on this data, it is possible to predict failures, anticipate maintenance, or plan corrective action in response to certain irregularities in real time [121,122].

Most of the application cases of DT in maintenance concern optimizing maintenance decisions [123]. The condition of the actual facility is predicted so that an appropriate maintenance plan can be selected. Monitoring the condition of the facility during the operational phase enables DT to plan maintenance more effectively. The differences between traditional and DT-based predictive maintenance are described in [120]. The use of DT in maintenance also contributes to technical systems’ reliability, efficiency, and safety [107,124,125,126,127].

**Figure 6:**
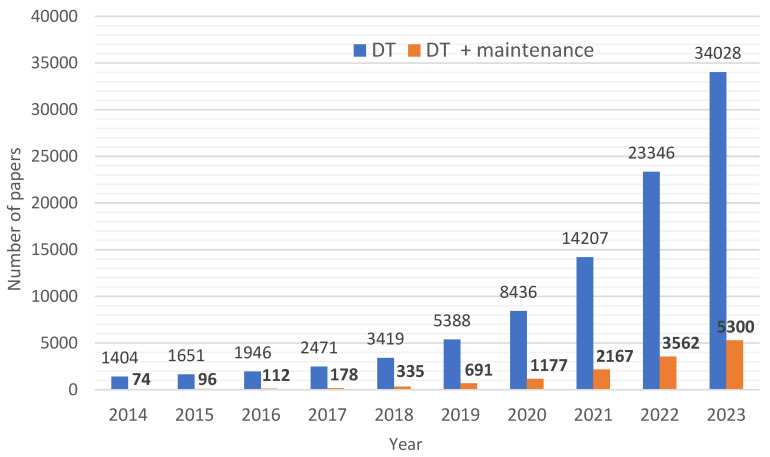
Publications from 2014–2022 that were published in the Scopus and Web of Science databases and included the term DT and combinations of the terms DT and maintenance. Source: own contribution.

The confirmation of increasing interest in DT implementation in the maintenance fields may be the analysis of the number of published papers per year. An initial study was performed based on the data from two databases, Web of Science and Scopus, in July 2024. The search process was based on using two keywords, “digital twin” and “digital twin AND maintenance”, and searching within all fields. The results are presented in Figure 6. Analyzing the graph, we may state that since 2018, there has also been an increasing trend in the number of publications that describe the link between DT and maintenance. Comprehensive reviews of digital twin use in maintenance areas, presented in, e.g., refs. [118,119,122,128,129], confirm this conclusion.

The fundamental nature and wide-ranging applications of digital twin (DT) technology, particularly in maintenance, highlight the necessity of examining its role in the operation and maintenance of transportation systems. The transportation sector encompasses various branches and addresses numerous issues requiring a systematic and organized approach. Therefore, it is essential to evaluate how the current review of digital twin technology, focusing on maintenance, emphasizes its significance in transportation system operations. This examination aims to ensure that the contributions and potential of digital twin technology in enhancing maintenance practices within transportation are appropriately highlighted and understood. Additionally, the objective is to identify the main research trends, knowledge gaps, and future research directions in this field, providing a comprehensive overview to guide further studies and advancements.

### 2.3. Digital Twin in Transportation Systems

The digital revolution has led to the development of intelligent transport systems technology. This has resulted in the widespread deployment of sensors in transport networks. These sensors provide real-time access to data that can be the basis for communication with a virtual model. This, in turn, enables the use of DTs in transport systems. Indeed, with the large quantity of data collected, DTs can potentially improve the transport sector. As a result, a DT can currently operate, control, or analyze existing/designed transport systems [130].

A preliminary analysis of the literature shows that, from 2019 onwards, a marked increase in the number of publications can be observed in the context of the design and implementation of the concept of digital twins to ensure the operational continuity of transport systems [99]. In some publications, the term transportation digital twin (TDT) appears. As TDT is in its infancy, it is, therefore, difficult to find a single, universally accepted definition of the concept [131]. As in the general definition of a DT, a TDT can be defined as a digital representation of a transport system’s physical elements that react to real-time changes. Both transport assets and connected services, even those that are not transport-related, are digitally mapped. The term TDT is not yet widely used, and it is most common in the literature to find a description of DT for specific applications of this technology in selected transport systems.

This concept finds application in all branches of transportation. It is currently used in maritime transport for predicting potential failures, optimizing fleets, ports, and terminals, and for comprehensive supply chain optimization [132,133]. In air transport, DTs can be utilized to control airport transportation systems [134]. The land transport sector is also widely described in the literature. DTs are applied in both long-distance transport (rail and road) and internal transport. In rail transport, authors propose using DTs to monitor railway switches, increase railway network capacity, and general management in the sector [135,136,137]. Road transport is another widely discussed application area for DTs, where it is used for optimizing traffic conditions, planning urban transportation, calculating recommended vehicle speeds, controlling traffic signals, visualizing possible scenarios, and enhancing road safety [138,139,140,141,142]. DT is most commonly proposed in internal transport for planning, optimizing transport processes, and vehicle route planning [143,144,145].

Although transportation infrastructure is gradually adapting to new technologies, the application of digital twins in transportation engineering is currently in its early stages [138,146]. The main goal of using DT in transportation is to enhance the safety and mobility of transportation systems [131]. The first review articles in this area appeared in 2020 and focused on selected application areas. The first review article on the use of DTs in transportation was published in 2020 by R. Phanden et al. [106]. This article describes the application of DT technology in aviation, robotics, and manufacturing. The authors focused on DT simulations and analyzed eleven aviation-related works, two on robotics and five on manufacturing. This analysis involved presenting how simulations and DT were utilized in each publication.

The article [101] pertains to internal transport. The authors presented five main trends that contemporary research on DT in internal transport systems focuses on. A broader analysis included 34 publications. This analysis focused on the use of DT in internal process optimization. It was noted that DTs are mainly built to increase the physical object’s efficiency and respond better when disruptions occur due to random events. The authors in [147] proposed a systematic review of the literature on current applications of DT in railway and road networks. The analysis results indicated that most DT applications in this sector are concerned with operation and maintenance. Another review article is dedicated to the maritime transport sector [148]. The DT of a ship is most often used for maintenance planning, failure prediction, and process optimization on the ship. However, the authors emphasize that real-time communication with the physical object is the biggest challenge in using a ship’s DT. In [131], publications focusing on transport safety and mobility were analyzed. The authors also proposed a concept of DT for transportation systems. Kaiblinger et al. presented the current trends in DT development in production logistics in their publication [149]. In [130], the authors focused on analyzing the potential applications of DT in electric autonomous vehicles. The analyzed articles concerned battery charging, driver experience, and vehicle monitoring and control.

**Figure 7:**
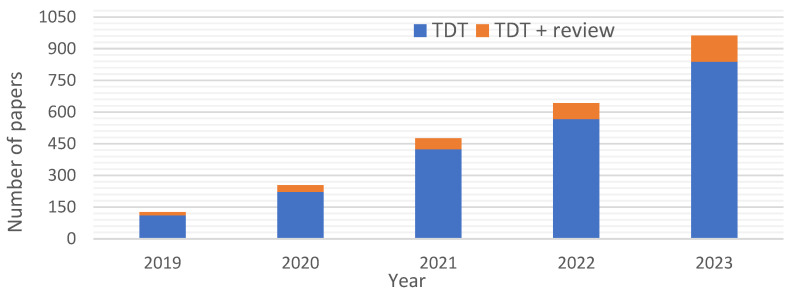
A number of publications and review articles in Scopus and Web of Science databases between 2019 and 2023 related to digital twins in transport systems. Source: own contribution.

Again, the confirmation of increasing interest in DT implementation in the transportation sectors may be in analyzing the number of published papers per year. An initial analysis was performed based on the data from two databases, Web of Science and Scopus, in July 2024. The search process was based on using two keywords, “digital twin AND transport” and “digital twin AND transport AND review”, searching within all fields. The results are presented in Figure 7. A summary of reviewing articles that focus on DT use in transportation systems is presented in Table 2. 

**Table 2:** A summary of recent papers focusing on providing a literature overview in the area of the digital twin concept used for transportation systems.

Ref.	Publication Year	Research Objectives	Papers Analyzed	Transport Sector	Digital Twin	Digital Shadow	Digital Model	Level of Analysis (System/Process/Object)
[106]	2021	Review of simulation-based DT and DT-based simulation models proposed for aerospace, manufacturing, and robotics	18	Aerospace	x	x	x	Object/process/system
[101]	2021	Overview of the academic research on the DT applied in internal transport systems of which inherent attributes are flows of materials and accompanying information.	110	Internal transportation	x			Object, process
[41]	2021	Review of technology, development, and types of digital twins, as well as possibilities of their application in logistics	n/a	Logistics (internal transportation)	x			Object/system
[130]	2022	Review of research works on DT technology for intelligent transportation systems focusing on the use of DTs in electric mobility and in autonomous vehicles	38	Land transportation (road)	x			Object, system
[148]	2022	Definition of the term “digital twin of a ship”; review analysis of developed digital twins for ships	19	Maritime transport	x			Object
[147]	2022	Presentation of the current scope of DT applications in railway and road networks with particular focus on sustainability and resilience	20	Land transportation (road, rail)	x	x		Object
[131]	2022	Reviewing of literature on transportation-related DT systems, presenting a reference architecture and framework for TDT systems focused upon safety, mobility, and environmental aspects, and identifying the challenges that arise from the requirements of such TDT systems	21	Land transportation (road)	x	x	x	Object, system
[149]	2022	Presentation of current DT development trends in production logistics	28	Land transportation (road)	x			Object/system
[150]	2022	Study of the application of BIM and DT in the transportation industry	493	Land transportation (road, rail), air transportation	x			Object
[5]	2024	Examination of the potential of digital twins in urban risk management, specifically in addressing disaster risks and enhancing resilience in urban environments	17	Land transportation (road)	x		x	Object, process, system

In summary, interest in the digital twin concept is steadily growing in relation to transportation systems performance, as confirmed by the data in Figure 6 and the review articles discussed in Table 2. DTs have a wide range of applications in transportation systems across all transport branches (air, maritime, and road transport). This technology is increasingly important in this area, contributing to improved efficiency, safety, and sustainability. On the other hand, the growing customer demands placed on transportation companies are becoming more diverse. Customers expect quick and timely delivery of goods and high-quality service, safety, and flexibility in adapting services to individual needs. High customer demands mean that transportation companies must ensure the efficient operation of their fleets and quality of services provided at every stage of the transportation process. This requires not only the implementation of quick and efficient customer service procedures but also the possession of a modern vehicle fleet with effective maintenance management and quick response in emergencies. These challenges can be addressed using DT, which can be applied to predict failures, anticipate maintenance, and plan repair actions based on real-time data. 

Additionally, while previous reviews have explored DT applications within specific transportation sectors or broadly within operational and maintenance contexts, this paper offers a novel contribution by providing a comprehensive synthesis of DT applications across multiple transportation domains with a focused emphasis on in-house logistics. Our review addresses two critical gaps in the current literature:

- Lack of comprehensive reviews: Although there is a growing body of work on DTs in various sectors, there is a notable absence of reviews that integrate insights across different transportation branches specifically in the context of operational and maintenance (O&M) practices. Most existing reviews either focus narrowly on specific transportation modes or broadly on DT applications without delving deeply into the unique O&M needs of internal logistics. This paper fills this gap by offering a detailed overview and critical analysis of how DTs can enhance O&M across diverse transportation sectors with particular importance of logistic systems.
- Insufficient focus on in-house logistics: The application of DTs in the maintenance of transportation systems, particularly in-house logistics, remains underexplored. Our review emphasizes this aspect, providing a structured framework that highlights how DT can address the unique challenges associated with internal logistics management. This focus on in-house logistics is a distinguishing feature of our work and represents a significant advancement in understanding and applying DT technology in this critical area.

## 3. Review Methodology 

The presented section outlines the main adopted assumptions and steps in the systematic literature review (SLR) adopted in this study. An SLR focuses on identifying, evaluating and interpreting all available research relevant to a particular research question, or topic area, or phenomenon of interest [151]. It is widely accepted that conducting an SLR is a fundamental scientific activity that follows a standard procedure for developing, conducting, and reporting processes [152,153]. 

**Figure 8:**
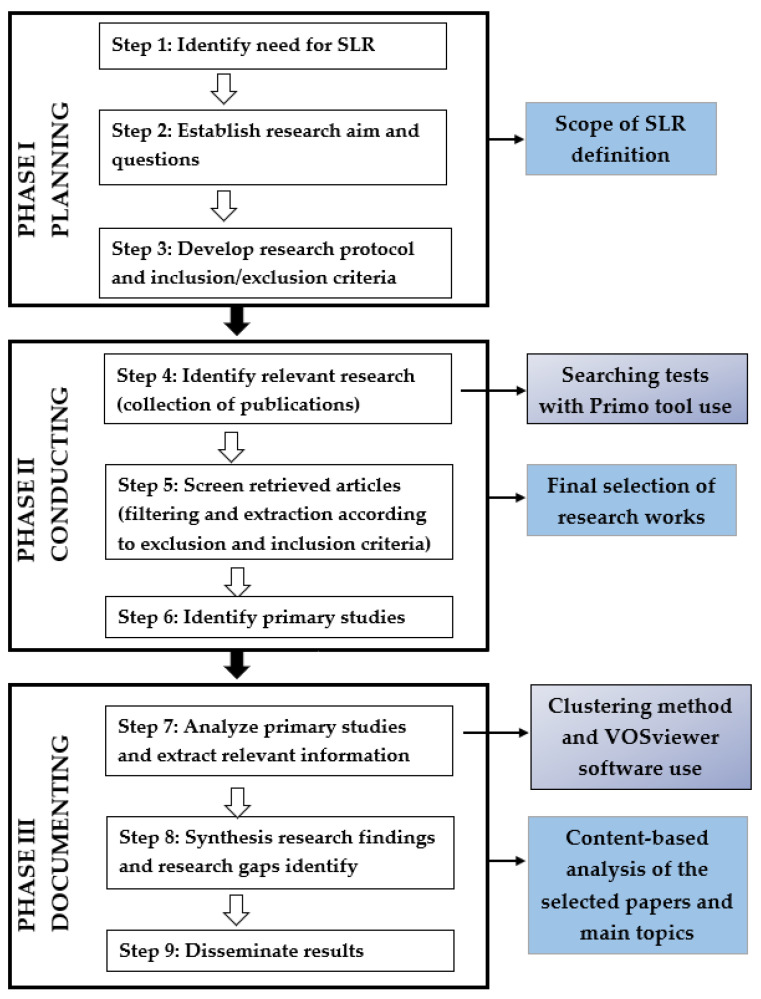
Research framework and methods/tools used for systematic literature review. Source: own contribution.

The main goal of the conducted review is to investigate the main research directions and gaps in digital twin modeling in the context of transportation systems operation and maintenance. The SLR was performed based on the established guidelines proposed by [152,154,155]. The basis for reporting systematic review conducted by the research team was the PRISMA guidelines [156,157]. The SLR process consists of 9 steps across three phases, namely, planning (3 steps), conducting (3 steps), and documenting (3 steps). It is illustrated in Figure 8. 

The next subsections discuss in detail the research work conducted in these three phases.

### 3.1. Planning of the SLR Study

In this section, the main steps related to the planning of this SLR study are presented. The motivation of this study is to investigate, classify, and synthesize the relevant literature on digital twins in the operation and maintenance of transportation systems based on the thematic overview of the primary studies. As an output of the first step of the planning process, the main objectives of this study are defined. As has been previously stated, they include (a) establishing the body of knowledge of transportation systems operation and maintenance performance in the context of digital twin use by identifying and classifying the extant research on this topic; (b) identifying the main knowledge and research gaps in this research area; and (c) identifying development trends and the opportunities for future research. To achieve these objectives, the main research questions are stated (Step 2): 

RQ1: What is the state of the literature on digital twin use in transportation systems operation and maintenance between 2012 and 2024?

RQ2: What are the main research and knowledge gaps in DT use in transportation systems operation and maintenance, especially in the context of in-house logistics? 

RQ3: Which aspects of DT modeling require further advancement to address future challenges in transportation systems?

RQ4: What scope should the framework for digital twin for maintenance management of transportation systems have?

The definition of the research questions was preceded by an extensive analysis of the issues addressed in the literature in the context of DT modeling, DT use in maintenance, DT use in transportation systems, and DT use in production and industry sectors according to the theory background, presented in Section 2. The identified research gap clearly indicates the need for research to develop a framework for digital twin-based maintenance management of transportation systems in the context of in-house logistics operations. In addition, the defined research questions and preliminary analysis of the available literature provided the possibility to determine the research framework for this study, relevant tools and methods to be used, and the main inclusion and exclusion criteria (Step 3).

### 3.2. Conducting the SLR Study

The second phase of the performed methodology includes steps 4, 5, and 6, which are discussed in detail below. 

#### 3.2.1. Collection of Publications for Review 

First, the literature-searching process was carried out. It was based on the use of the multi-search tool Primo [158]. The Primo tool searches a library’s collection of resources based on keywords and uses a range of filters to refine one’s analyses. Based on this, we can create search strategies based on resources from different scientific databases, such as e.g., Scopus, Web of Science, ScienceDirect database, Elsevier, Wiley, and Springer publisher databases. The literature search was conducted between 17 June and 17 July 2024.

The search string covered English search terms used in various combinations applying a Boolean operator, AND and OR. The search query was based on keywords related to digital twin, maintenance-related, and transportation-related aspects. The maintenance-related keywords were identified based on review papers [122,129,159,160,161], whereas transportation-related keywords were defined according to review papers [6,99,101,162,163]. In addition, the keyword selection process was designed to encompass both contemporary and historical terms relevant to DT technology in transportation systems operation and maintenance. As a result, we took into account the relevance of keywords to the research scope and historical context. We also applied an initial broad search strategy with a review of related terms (e.g., “cyber-physical systems”, “simulation-based models”, “virtual models”) before the final definition of the keywords. 

The final selected keywords were determined to provide the widest possible coverage of the digital twin-based research in the context of operation and maintenance and transportation systems. The final search engine includes the following: 

  (ALL = (digital twin)) AND (ALL = (maintenance OR maintenance management OR fault OR diagnosis OR prognosis OR condition monitoring OR predict OR diagnostic)) AND (ALL = (transport OR transportation OR robot OR warehouse OR mobile OR railway OR aircraft OR vehicle OR land OR logistic OR forklift OR intermodal OR internal))


Based on the presented criteria, the initial search allowed for the identification of 2509 scientific papers, which were later analyzed in the screening process (Section 3.2.2). 

#### 3.2.2. Screening of Collected Publications 

The screening process allowed for the identification of papers relevant to full-text analysis. First, the studies were limited to those published between 2012 and 2024 to ensure recent and relevant advancements were included. 

To ensure a thorough and accurate evaluation of each study’s relevance to the topic of digital twin (DT) applications in transportation systems’ operation and maintenance, we imposed two key inclusion criteria: full-text availability and publication in English. The availability of full text was crucial for an in-depth assessment of the study’s content and its alignment with our thematic focus. This allowed for us to thoroughly review and evaluate the methodologies, findings, and relevance of each paper. Additionally, English-language publications were selected to facilitate comprehensive understanding and consistent interpretation of the research, ensuring that the studies could be accurately assessed for their contribution to this review’s objectives.

Based on these inclusion criteria, 124 papers were excluded from further analysis. 

The screening process had the purpose of filtering out papers that were not related to the main topic. Therefore, a two-step analysis was performed. First, the records were scanned by title and abstract by all authors. Studies were included if their abstracts indicated a focus on DT applications in transportation systems, operation, and maintenance. Later, we compared team members’ opinions at a research team meeting. In case of discrepancies in assessing the paper’s suitability, the team members decided to include the questionable articles in the full content analysis. After this operation, 1072 papers remained in the second step. Before a full-content analysis, duplicate records and review papers were removed. 

In the second step of the screening process, the authors examined the papers in the full-text research. The main aim was to assess their relevance to the investigated thematic area. Papers were evaluated based on their contributions to understanding DT applications, methodologies used, and their implications for maintenance and operational practices in transportation systems. As in the first step, the research teams made the evaluation individually. Later, at research team meetings, we compared team members’ opinions. In case of discrepancies in assessing the paper’s suitability, the team members focused on a more detailed analysis of the full document. As a result, 730 papers were excluded for specific reasons. For example, the studies that describe maintenance issues, e.g., medicine applications, were excluded.

#### 3.2.3. Primary Studies Identification 

After the screening process, 201 publications were selected for further qualitative and quantitative analysis. A cross-sectional review of the identified papers was also conducted to ensure we accumulated a relatively complete census of the relevant literature [164]. As a result of the analysis carried out, it was confirmed that the identified publications provide a complete state of the art in the research area analyzed.

**Figure 9:**
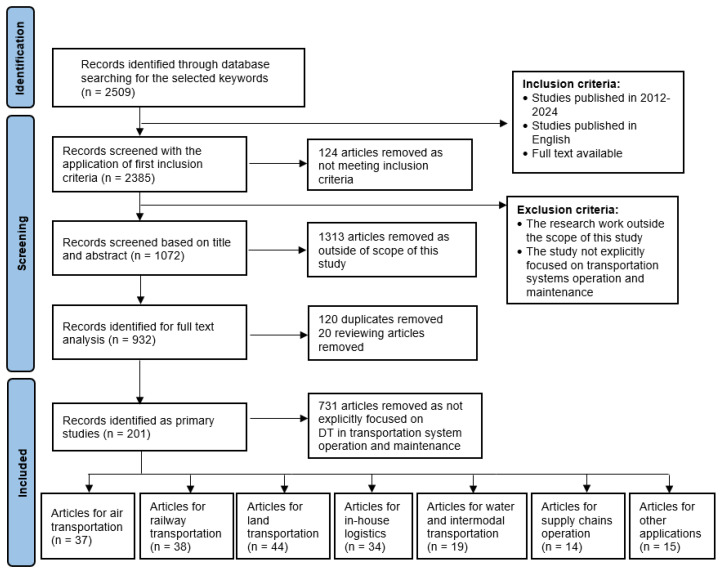
PRISMA-based flowchart of systematically selecting relevant studies in the analyzed research area. Source: own contribution based on [36].

Figure 9 represents the flow diagram of the selection of studies according to PRISMA statements. The PRISMA checklist is available in Supplementary Materials (Table S1).

### 3.3. Documenting of the SLR Study

This phase includes steps 7, 8, and 9 of the conducted SLR study. A bibliometric analysis was performed as part of the performance in step 7. 

Bibliometrics is a branch of scientometrics that uses mathematical and statistical methods to assess the performance of scientific activities. The bibliometric analysis allows for us to study the networks formed around the most representative keywords. It presents how citations, scholars, affiliations, counties, and publications indicate the importance of specific topics in the field of research. At the same time, we can see a noticeable increase in interest in bibliometric studies in science (see, e.g., refs. [160,165,166,167]).

Following the analysis, the selected articles were stored, documented, and classified using the Mendeley reference manager [168,169]. The primary content-based analysis was performed using MS Excel Professional Plus 2019 software and VOSviewer ver. 1.6.18 software [170]. The main results are presented concerning, among others, the authors’ location, publication time, or number of citations. The citation overview was made based on the Google Scholar database. 

In addition, based on [171], VOSviewer is a program developed for constructing and viewing bibliometric maps that can be examined in full detail. The quantitative summary includes an analysis of the occurrence of trends. In addition, distribution by year and publication source was performed. Following the functionality of VOSviewer software, we constructed bibliometric maps and examined them in detail. The distance-based bibliometric maps that were created focus on keyword co-occurrence and relations between main authors. The results are presented in Section 4. 

Step 8—synthesis of research findings was performed. The obtained outputs were discussed in relation to the four defined research questions. 

The last step is connected with the development of this study with a clear definition of its limitations and directions for further research. The results of step 8 and 9 are presented in Section 5. They constitute the basis for developing the framework for DT-based maintenance management of transportation systems in the context of internal transportation performance. 

## 4. Systematic Review Analysis Results

This section includes the results of the conducted systematic review according to the defined research methodology (Figure 8). 

### 4.1. Bibliometric Analysis

In the first step, a bibliometric analysis of already-selected publications for further research on the topic of DT use in the transportation sector was carried out. A total of 201 publications from the seven subject areas analyzed were accepted for detailed analysis. The largest number of articles (44 papers) was in the area of the link between DT and land transportation (road). The number of analyzed publications in the other areas is as follows: in the area of railway transportation—38 publications; air transportation—37 publications and in-house transportation—34 publications; water and intermodal transportation—19 publications; and in the area of supply chains operation, 14 publications. To the last group, “other applications”, 15 publications were assigned. 

**Figure 10:**
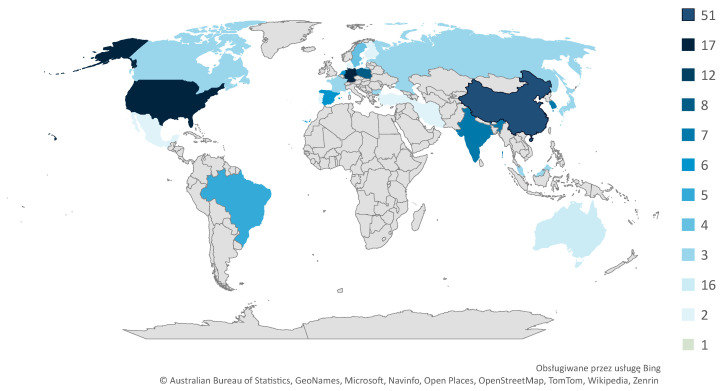
A number of papers by the location where the investigated study took place.

The analysis of the authors’ and scientific centers’ origins was also a part of bibliometric analysis. The carried out analysis shows clear differences in scientific activity around the world. China definitely dominates in terms of the number of publications, as the number of papers coming from this country accounts for 25% of all items analyzed. There is also strong scientific activity in the United States and Germany, with 17 papers each. Other countries have varying levels of publications, including Australia (16 papers), England (12 papers), India (8 papers), Spain and Italy (7 papers each), South Korea and Russia (6 papers each), Poland (5 papers), and Brazil (4 papers). The regions of origin of the authors of the analyzed publications are shown in Figure 10. Analyzing the scheme in terms of continental division, the largest number of articles comes from Europe, accounting for 49% of all publications. Asia also shows high publication activity in the area, with 37% of the analyzed articles coming from there. In contrast, 10% of publications come from North America, 8% from Australia, about 3% from Africa, and less than 3% from South America.

The dominance of certain countries in the number of publications related to digital twins (DTs) may indicate increased research intensity as well as investment in DT technology in these regions. Countries such as China, the United States, and Germany have been investing in scientific research and technological development for many years. Digital twins are an advanced technology that requires significant financial resources, research assets, and access to advanced infrastructure, which is possible in these countries due to well-developed research and industrial ecosystems, as well as strong governmental support. Government support is reflected in numerous research and innovation funding programs aimed at implementing strategies such as “Industry 4.0”.

The predominance of publications from Europe and Asia may lead to a limited perspective, focusing primarily on issues, solutions, and research contexts characteristic of these regions. A lesser representation of research from other parts of the world, such as Africa or Latin America, may result in the omission of specific challenges and opportunities related to digital twins in various cultural, economic, and technological contexts. Additionally, research on digital twins often concentrates on sectors that are highly developed in the leading countries, such as manufacturing, automotive, or advanced technologies. Other sectors, such as education, agriculture, or public services, may be less explored, leading to gaps in the literature and potential underestimation of DT applications in these areas. Moreover, most scientific publications are in English, which may exclude important research conducted in other languages. This can lead to the oversight of significant findings and perspectives from research conducted in countries where English is not widely spoken.

**Figure 11:**
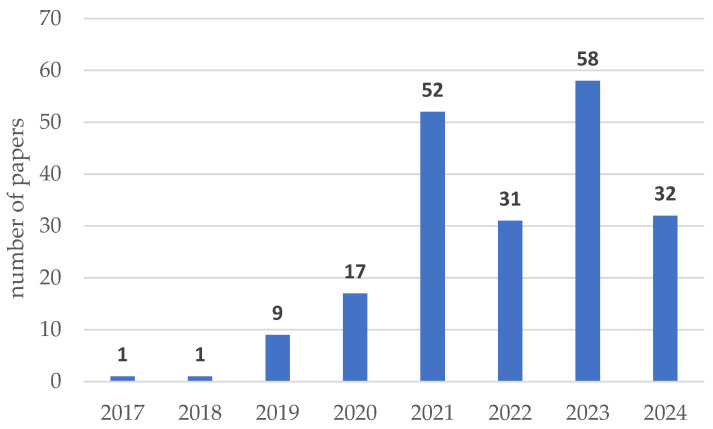
Distribution of publications by year.

This review brings together 201 publications that were published between 2017 and 2024. Figure 11 illustrates the distribution of the publications according to their publication year. As we can see, a significant number of publications (173) were published between 2021 and 2024. In 2024, the analysis includes articles published until June, accounting for 59% of the articles published in 2023. Given that the data cover only the first half of the year, it can be predicted that the total number of publications this year may be higher than the previous year. 

**Figure 12:**
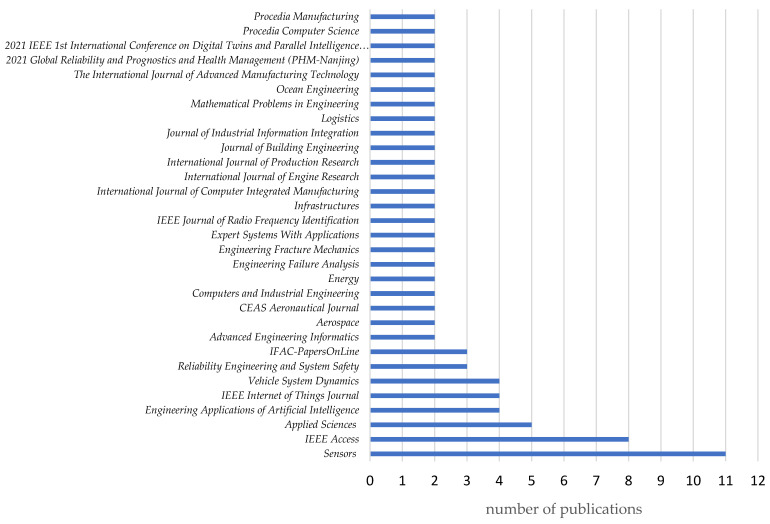
Number of publications with journal sources (for journals with at least 2 published articles out of the 201 articles analyzed).

In addition, the studied articles were published in 142 journals. Figure 12 shows the graph of a list of journals in which at least two articles were published in the area under study. Eighty-eight articles from 31 journals were analyzed. During the analyzed period, the largest number of publications appeared in the journal *Sensors* (11 articles). The second highest number of publications in the studied area was in the journal *IEEE Access* (eight publications). *Applied Sciences* published five articles, and *Vehicle System Dynamics* and *Engineering Applications of Artificial Intelligence* published four articles each. In comparison, *IFAC-PapersOnLine* and *IEEE Internet of Things Journal* published three articles each. The remaining publishing outlets published two articles each on the topics under study.

Dominant journals (such as *Sensors*, *IEEE Access*, and *Applied Sciences*) promote an interdisciplinary approach to scientific publications. This makes them ideal venues for publishing research related to digital twins (DTs), which, as a technology with a broad range of applications, integrates aspects of engineering, computer science, and systems management. The primary focus of articles accepted and published by *Sensors* revolves around sensors and monitoring systems, which are crucial for the development of DTs. Consequently, it is natural that the highest number of publications in the analyzed area has appeared in this journal. Additionally, *IEEE Internet of Things Journal* is dedicated to new technologies related to IoT, which is an integral component of digital twins. A DT utilizes IoT for real-time data collection, making this journal a fitting platform for research in this field. *Applied Sciences* and *Vehicle System Dynamics* are journals with strong connections to industrial applications and applied engineering. DTs are significant for industries, especially in areas such as automotive, manufacturing, and engineering systems, which explains their presence in these journals.

The high number of publications in these journals may also indicate their prestige and broad visibility within the scientific community. Authors may prefer to publish their work in widely read and respected journals, which enhances the impact of their research on the development of digital twin technologies.

**Figure 13:**
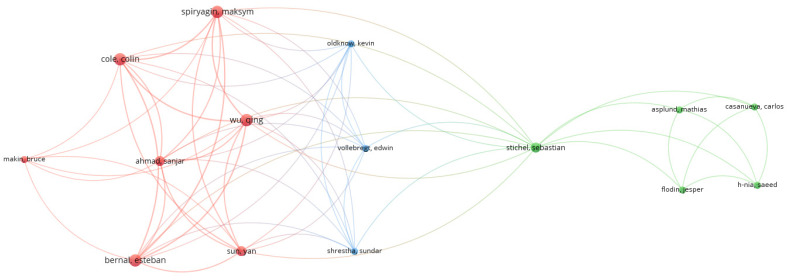
The largest set of connected items based on co-authorship links. Source: own development using VOSviewer software [170].

**Figure 14:**
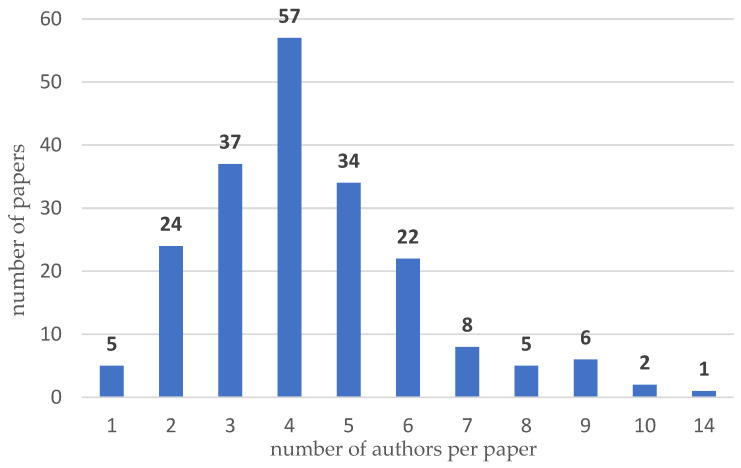
Distribution of publications per number of authors.

To supplement the conducted analysis, a co-occurrence of authors was investigated using VOSviewer software and Excel software. For the selected papers, 81 authors were identified. In Figure 13, results are presented for 15 authors who had the largest set of co-authorship links. The largest set of links (18) has four authors: Bernal Esteban (co-author of three papers [172,173,174]), Cole Colin and Maksym Spiryagin (co-authors of four papers [172,173,174,175]), and Qing Wu (co-author of four papers) [172,173,174,176]. All authors are from Australia. In addition, the distribution of publications per number of authors per paper is given in Figure 14. This research indicates that multi-author articles predominate, especially those written by teams of three to five authors. Single-author articles are in the minority, suggesting that research papers are more often carried out in teams than individually. As can be seen, very large research teams (more than five authors) occur less frequently in the analyzed sample of publications.

**Figure 15:**
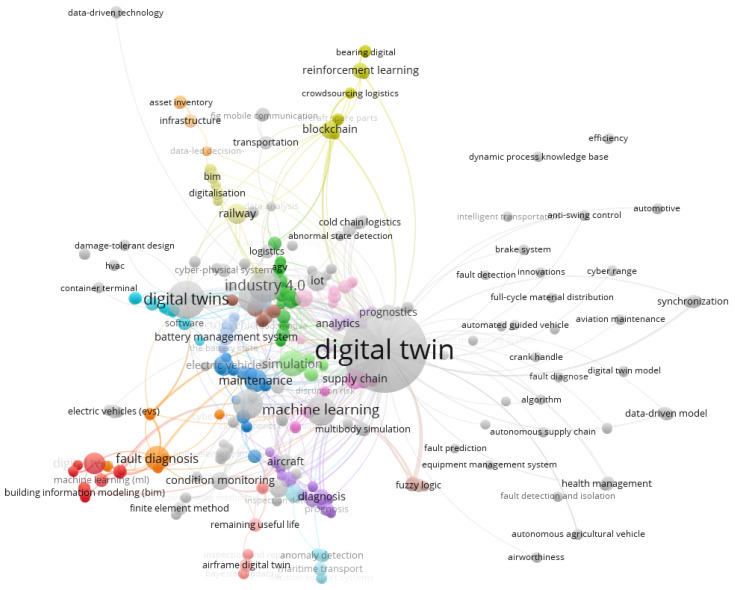
Mapping of the keywords that have occurred in the selected publications at least once. Source: own development using VOSviewer software [170].

The last part of the bibliometric analysis was a keyword co-occurrence analysis based on using VOSviewer software. The initial study focused on the keywords that occurred in the publications at least once. As a result, 609 keywords were identified for the selected papers (Figure 15). 

The results present the used keywords in 67 clusters. The most used words were digital twin (121 links), digital twins (20 links), and Industry 4.0 (15 links). Also, the words “machine learning” and “predictive maintenance” occurred frequently (12 links with total link strength equal to 76). One of the largest clusters of publications (27 items, red one in Figure 15) underscores the significance of digital twin technology integrated with machine learning and deep reinforcement learning. It emphasizes the transformative potential of digital twins in various fields, from building management and logistics to transportation and aerospace, highlighting their role in efficiently monitoring, modeling, and optimizing complex systems. The second largest cluster of publications (27 items, green one in Figure 15) revolves around applying digital twin technology to mobile robots, automated guided vehicles (AGVs), agile manufacturing, supply chain management, and vehicles. Central to these studies are various modeling methods, including virtual models, advanced simulation, fuzzy logic, genetic algorithms, graph theory, and trajectory optimization, which are employed to enhance the effectiveness and precision of these systems. The third interesting cluster of publications (26 items, blue one in Figure 15) focuses on applying digital twin technology in maintenance, repair, risk analysis, and decision-making. Central to these studies are various methodologies, including time series analysis, stochastic optimization, inspection processes, and integration with cyber–physical systems, electric vehicles, battery capacity, and battery management. 

**Figure 16:**
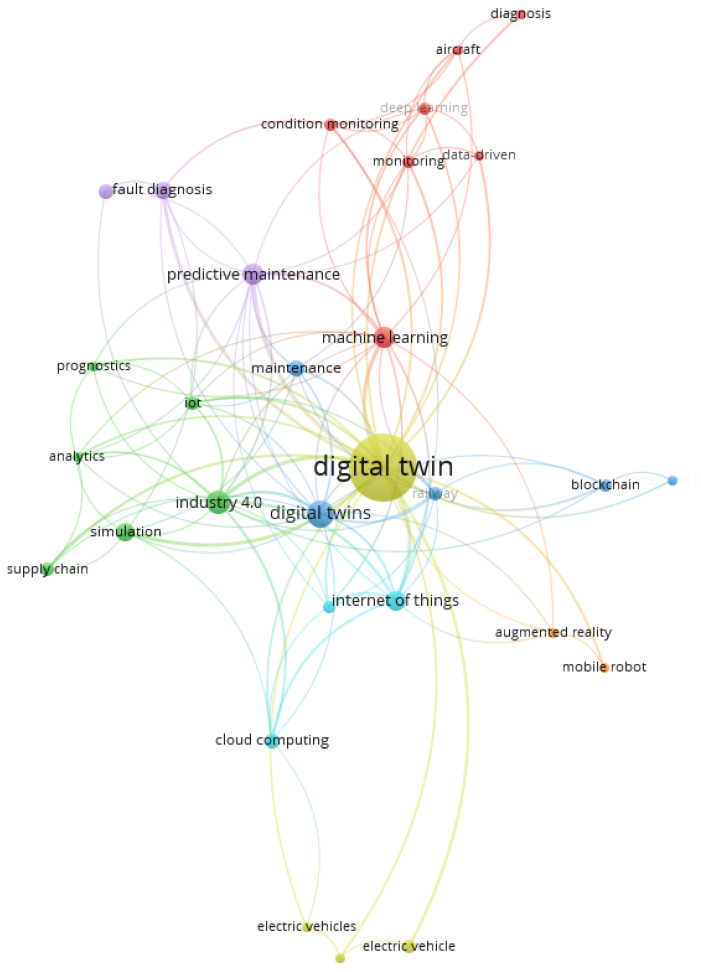
Mapping of the keywords with the largest occurrence. Source: own development using VOSviewer software [170].

A more detailed analysis was focused on the keywords with the largest occurrence sets (Figure 16). The results present the 30 most frequently used keywords in seven clusters. 

The largest cluster (seven items, red one in Figure 16) is strictly connected with applying digital twin technology in the aviation industry and the problems of condition monitoring, diagnostics, and predictive maintenance. Central to these studies are data-driven approaches and advanced techniques such as machine learning and deep learning, which enhance the accuracy and efficiency of monitoring and diagnostic processes.

The second cluster of publications (six items, green one in Figure 16) centers on the application of digital twin (DT) technology within the context of Industry 4.0, focusing on analytics, the Internet of Things (IoT), prognostics, simulation, and supply chain management. This cluster highlights the transformative impact of digital twins in modern industrial landscapes, where interconnected systems and data-driven decision-making are paramount.

The third cluster (six items, blue one in Figure 16) focuses on the innovative convergence of digital twin technology with blockchain, maintenance strategies, reinforcement learning, and railway systems. This multidisciplinary approach highlights the potential for enhancing railway operations’ efficiency, security, and reliability through advanced digital solutions. It highlights how these technologies can work together to provide secure, transparent, and optimized solutions for managing complex systems (including railway systems). Integrating digital twins with blockchain ensures data integrity and trust, while reinforcement learning enhances adaptive maintenance and operational efficiency, ultimately leading to more reliable and efficient services. 

At the end, the fourth cluster (four items, yellow one in Figure 16) explores integrating digital twin technology with battery management systems in the context of electric vehicles (EVs). This interdisciplinary research highlights how digital twins can significantly enhance the performance, efficiency, and reliability of battery systems in electric vehicles.

The performed bibliometric analysis introduces the comprehensive content-based analysis, which is carried out in the next section. 

### 4.2. Content-Based Analysis 

**Figure 17:**
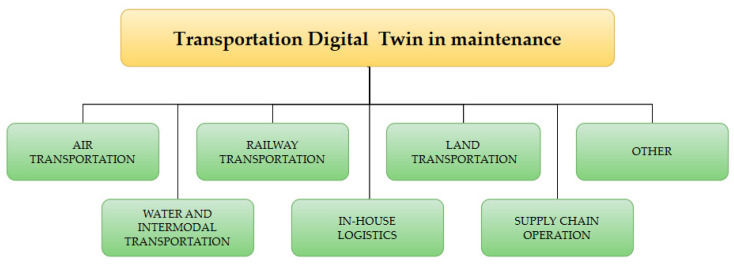
The main areas in the context of the transportation digital twin in maintenance. Source: own contribution.

As a result of the conducted research, seven core research areas were defined, which have been most extensively developed over the last seven years (Figure 17).

#### 4.2.1. DTs in Air Transportation

**Figure 18:**
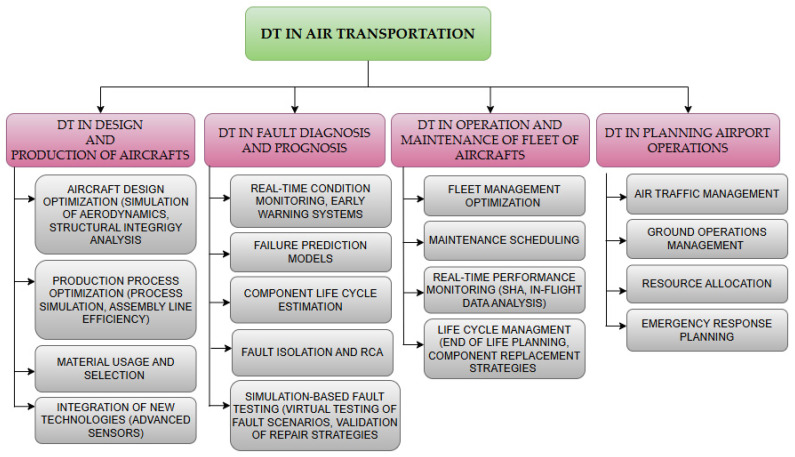
The main areas of DT implementation in the aviation industry. Source: own contribution.

Due to the rapid development of the aviation industry, the expansion of aircraft fleets, and the design of increasingly complex operational processes in the air transport sector, a need to utilize advanced technologies that support the maintenance management of aviation systems has arisen. With the growing availability of data from various aviation processes, a technology that has begun to be employed in recent years to achieve these goals is the digital twin. Given the need for continuous monitoring and improvement of complex aviation systems, DTs are frequently used by aircraft manufacturers, airlines, and airport operators. In the aviation sector, the application of DT technology is broad. An overview of the possibilities of using DTs for the aviation zone can be found in [177,178]. Meanwhile, in [88], the development and goals of DTs for airports are presented. In [87], the challenges associated with digitization and the implementation of DTs in the aviation industry are discussed. Figure 18 shows the main areas of DT application in the aviation sector.

DT has found its application in the aviation sector in producing flying objects, providing numerous benefits for manufacturers, such as increased efficiency, optimization of production processes, and design process improvement. The general structure for intelligent planning of processes related to the production of aviation parts based on the DT concept is described in [179]. The use of DTs for the ground control system of an aircraft’s nose landing gear to assess the accuracy and integrity of the steering angle estimation for various control algorithms is presented in [180]. It has been demonstrated that for these purposes, the soft computing algorithm exhibits higher accuracy compared to least squares algorithms. In [181], the specification of DT for a shared workspace for humans and robots, where electromechanical actuators are mounted on an aircraft wing, is presented. A DT of an aircraft’s power electronics cooling system (PECS) for the optimal placement of sensors in this system was introduced in [182].

DTs play a significant role in monitoring the technical condition of aircraft and in real-time maintenance forecasting and optimization. This capability allows for quick responses to emerging anomalies and minimizes the risk of aircraft failures, thereby reducing airport downtimes. In [183], a method for analyzing data from measurement devices installed on board an aircraft using a DT is presented. The DT of an aircraft engine for fault detection, isolation, and identification is described in [184,185,186,187,188,189]. In [190], a diagnostic algorithm for the electrical power system in an aircraft was developed to detect faults and their root causes, where one of the creation stages involves developing a DT of the electrical power system. Ref. [191] discusses the use of DT for diagnosing and forecasting the technical condition of aircraft electrical equipment. In [192], the authors focus on the use of DT in monitoring the power system of civil aircraft. Meanwhile, ref. [193] describes the application of smart devices in aviation maintenance utilizing virtual reality (VR) and a DT. The authors of [194] designed a graphical user interface for a system using an aircraft DT and augmented reality glasses for its maintenance and repair. In [195], the DEVOTION methodology for DT development was proposed. The authors developed an extensible DT platform to ensure the electrical and electronic systems for space launch vehicles are secure. The authors of [196] developed a DT-based system to investigate the problem of dynamic resource allocation for the communication needs of vehicles assisted by unmanned aerial vehicles (UAVs) and reconfigurable intelligent surfaces (RISs). This approach reduced energy consumption and minimized transmission errors in variable environments. In [197,198], discussions on how DTs can improve monitoring, damage assessment, and decision-making in aircraft design, maintenance, and fleet management are provided. The authors of [199] proposed a modular DT architecture for aircraft supporting maintenance processes. In [200], an analysis of fatigue life prediction for an electric motor shaft in an airplane was conducted using a DT of the rotor shaft in the electric motor to simulate the stresses encountered. A DT of the fan blade grinding process in an aircraft is presented in [201], where the DT was developed to study the required grinding parameters. In [202], an aircraft DT was introduced to determine safety and reliability. The article also describes the current state of knowledge on DT risk assessment modeling for critical fatigue areas. The DT of an aircraft’s nose wheel for optimizing maintenance processes was developed in [203].

Ref. [204] describes a simplified method for diagnosing faults in any aircraft system using a DT based on the Open System Architecture for Condition-Based Maintenance. This concept was tested on three different systems. In [205], a DT supported by a multiscale residual self-attention feature fusion network for diagnosing damage in hypersonic flight vehicles is presented. A method for characterizing damage using sensors from various locations was developed in [206] to predict the damage’s location, size, and orientation. This method supports the creation of aircraft DTs for diagnosing damaged structures. Determining the remaining useful life of aircraft maintenance parts using various data analysis methods combined with a DT is presented in [207]. In [208], a system for tracking aircraft spare parts throughout the supply chain and the structure of DT integration into the proposed system is proposed.

Additionally, Ref. [209] presents the development of a general model for managing the mobility of electric air vehicles. A DT is used here to simulate and optimize air mobility. Ref. [210] discusses the application of DTs for training deep reinforcement learning (DRL) models to enable the collective movement of multi-drone UAV systems, where DTs facilitate the rapid deployment of trained models to real UAVs.

#### 4.2.2. DTs in Railway Transportation

**Figure 19:**
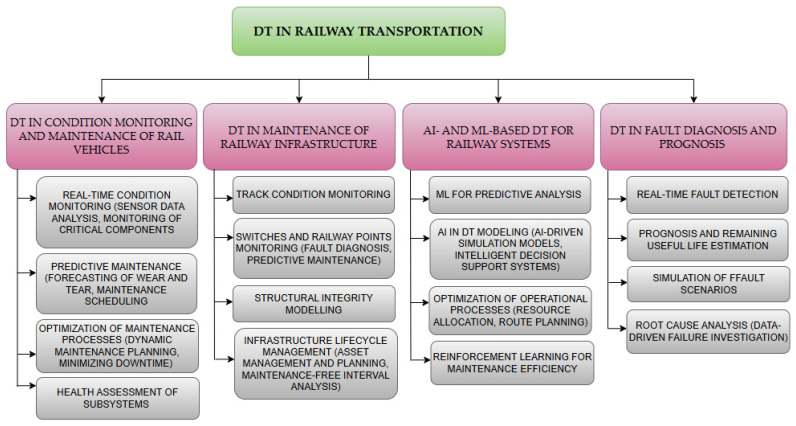
The main areas of DT implementation in the railway industry. Source: own contribution.

In recent years, significant progress has been made in the railway transport sector regarding digital transformation, particularly in implementing digital technologies and data utilization. For railway transport, DTs have great potential in supporting the management of railway infrastructure, operational processes, and passenger safety. Figure 19 illustrates the identified main areas of DT application in the studied transport sector.

The railway sector is one of the areas where DT technology is most frequently used in the maintenance management of railway assets. This is confirmed by numerous publications summarizing recent developments in this area. For example, Ref. [211] describes an overview of the possibilities of DT technology and its implementation in transforming railway maintenance and digitizing railway infrastructure and signaling. In [212], the activities of the Railway Technical Research Institute regarding the maintenance technology of the power system in electric railways are described. They propose DT technology to predict and assess the progress of degradation. A review of published research on the application of artificial intelligence (AI) in railway transport is presented in [213]. The authors highlight DT and the Internet of Things as the main technologies supporting AI. For other reviews, the authors recommend reading [214,215].

Most works in the area of DT application in the railway sector aim to monitor the condition of machinery and predict anomalies. The architecture for managing the condition of rail vehicles based on DT is presented in [216]. In [217,218], the use of DT is aimed at optimizing maintenance processes. Ref. [219] focuses on developing methods for diagnosing faults in rail vehicles using supervised machine learning. In [220], the use of a DT for simulating substitute wagons was proposed to reduce the computation time required to assess the safety of the entire train. The DT was used to eliminate data gaps, which enabled the creation of a dataset necessary for training the model. A DT for railway systems was proposed in [172] to reduce the risk of derailment. In [221], a DT of the heating, ventilation, and air conditioning system is described to monitor the condition of this system. In [222], a DT of a high-speed train bogie was presented to determine the operational conditions of such a bogie based on the analysis of vibration signals. The work also describes techniques for processing vibration signals. Ref. [223] presents the concept of a DT locomotive in the context of operation and maintenance management systems. In [224], a DT was applied in managing wireless networks of smart railways, where the DT enables these networks’ design, optimization, and lifecycle management. In [225], a DT was used to accurately predict the mass of solid particles in air filters in a passenger car, monitor filter wear, and identify faults.

Rail tracks play a crucial role in railway transport as a fundamental element of railway infrastructure. They perform essential functions necessary for the safe and efficient movement of trains. Therefore, in [226], a predictive model for the life of railway wheels and rail tracks is presented, which could be a part of a future digital twin of the railway system. In [227], a DT was used to correlate the assessment of visual conditions with mechanical performance. Meanwhile, in [173], a simulation modeling method was proposed for predicting rail surface damage based on the DT of a locomotive. In [174], a DT for simulating the dynamics of railway vehicles, particularly in the context of calculating surface damage to rails, was discussed. The assessment of the condition of railway tracks and their maintenance-free 3D reconstruction using a robotic system was presented in [228]. This research can be used as an autonomous generator of twin models, leading to improved railway maintenance DTs and intelligent railway infrastructure management. In [229], various approaches to modeling vehicle–track interactions and predicting rail damage were discussed, with DTs responsible for integrating and optimizing simulation models. Ref. [230] addresses the use of DT for simulating complex guided wave propagation in railway tracks under different conditions.

A railway switch is crucial in the railway transport system, enabling trains to change tracks. Switches must be well-maintained and functional to ensure the safe and reliable operation of the railway system. Regular maintenance and proper monitoring and control of switches are necessary to ensure the smooth flow of railway traffic and minimize the risk of failures. Consequently, several studies have focused on monitoring the condition of this specific element of railway infrastructure using DTs. In [231,232], DT-assisted fault diagnosis structures for railway point machines (or railway switch machines) are presented. Ref. [136] introduces a solution for monitoring temperature conditions and other atmospheric factors to make the DT of railway switches more accurately reflect reality. In [233], the use of a DT is described as part of a six-dimensional BIM model for a railway switch system.

Ref. [234] presents the application of reinforcement learning with a DT for optimizing the efficiency of railway infrastructure maintenance. In [235], the structure of a DT for urban railway transport and its implementation method are described. The concept of a DT for railway infrastructure is presented in [236]. To improve documentation accuracy and reduce human errors in the operation and management of railway infrastructure, ref. [237] discusses using a DT of a test railway track. Ref. [238] outlines a framework for designing and implementing DTs in railways. A smart railway station DT concept is described in [239]. Ref. [176] focuses on building an integrated model for data, models, and knowledge management to enhance railway project analysis and intelligent management. This enables dynamic linking and global searching for connections across extensive spaces. In [240], the application of the DT concept in railway control systems is discussed, with the authors primarily focusing on modeling and simulating railway signaling system elements. Refs. [241,242] demonstrate the use of DT for real-time monitoring of the structural integrity of railway bridges, allowing for early damage detection and optimization of maintenance actions. Meanwhile, ref. [243] describes the implementation of DTs for railway bridges in Germany, which is aimed at forecasting their condition and structural safety and optimizing maintenance efforts. Ref. [244] presents the potential to improve passenger safety and pedestrian traffic management at train stations through DT as a decision-supporting tool.

In a DT, the integral acquisition of data is crucial, as it forms the foundation for assessing the condition of the object under investigation. Therefore, the authors in [135] presented a new approach based on Internet of Things technology for intelligent data acquisition to generate DTs in the railway industry. 

#### 4.2.3. DTs in Land Transportation 

**Figure 20:**
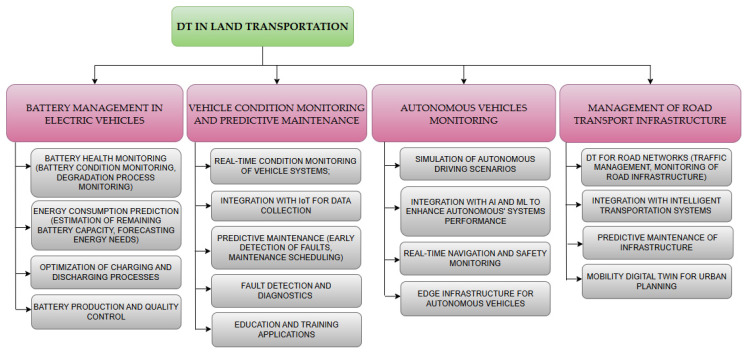
The main areas of DT implementation in land transportation. Source: own contribution.

In the case of land transport, digital twins can serve as a tool with significant potential, enabling effective and intelligent management of infrastructure and vehicles. Figure 20 illustrates the main areas of DT application in land transportation.

Many of the publications selected for this literature review focus on electric vehicles and battery management. The authors in [245] reviewed various DT applications in the electric vehicle industry. A review of publications describing the use of DTs for battery management was conducted in [97]. In [246,247,248], DTs for electric vehicle batteries are presented for energy consumption research, battery capacity estimation, and technical condition prediction. Meanwhile, DTs of electric vehicles for simulating realistic events that may occur in the real world is discussed in [249]. The authors concentrated on simulating electric vehicles’ charging and discharging processes during use. As a result of these simulations, it becomes possible to plan the charging of electric vehicles and to plan the placement of charging stations. DTs of electric vehicle batteries are described in [96,250,251]. In [96], the authors also proposed using a DT during the battery production stage. The DT of the production process facilitates the development of production lines and workshops to streamline the entire manufacturing process. Furthermore, a DT for electric vehicle battery management systems was proposed in [252,253]. In [254], a DT for an electric vehicle engine was developed to monitor and predict its condition. The authors in [255] addressed a broader area, focusing on DT for electric drives. Additionally, there is a study discussing the challenges of implementing electric vehicles. The authors in [256] highlight that a significant obstacle to the mass adoption of such vehicles is their complex internal structure, which makes the repair and maintenance of electric vehicles difficult and costly. To address this, the authors propose using a DT in servicing and repairing electric vehicles, significantly reducing time and associated costs.

DTs also support the development of connected and autonomous vehicles. Through simulations and machine learning, virtual testing and training for autonomous systems can be conducted, accelerating their development and introduction on the roads. DTs enable vehicle behavior analysis in various scenarios, allowing for the refinement of control algorithms and increasing road safety. In [257], comprehensive research on the application of DTs in autonomous vehicles is presented. The role of DTs as a development environment in improving the performance of autonomous systems supported by artificial intelligence is explored in [258]. Authors in [259] describe already-published applications of DTs in automated vehicles and intelligent transportation systems and the opportunities and challenges associated with applying DTs in automated vehicles. Ref. [260] discusses the methodology for creating and integrating smaller DTs for autonomous vehicle functions and examines the challenges related to their integration. In [261], a DT framework based on edge infrastructure for autonomous vehicles is presented. The topic of optimizing electric drivetrains in autonomous vehicles is addressed in [262], where the authors mention the potential use of DTs and virtual reality to monitor the condition of drivetrains. A comprehensive framework for the navigation of autonomous vehicles in infrastructure construction scenarios using DTs is outlined in [263]. Lastly, authors in [264] investigate the sources of unpredictability in the motion trajectory of DTs for connected vehicles.

Authors in [265] present the potential of DTs in vehicle condition management. They confirm that DTs can be utilized to assess the status of complex systems. The integration of DTs with predictive maintenance methods is discussed in [126]. A DT for fault detection is proposed in [266], where the authors suggest combining a DT and failure mode and effect analysis (FMEA) for online diagnostics of vehicles. In [267,268], a DT for diesel engines is developed to detect and predict anomalies in the driveline operation. The role of DTs in predictive maintenance is outlined in [269], focusing on the maintenance of automotive brake pads. The use of a DT for electric machines for educating electrical engineers is proposed in [113]. By analyzing data generated by the DT, students learn to diagnose potential failures or malfunctions of the machine. The development and validation of a DT for steel railway wheels, allowing for fatigue life prediction, is presented in [270]. Meanwhile, ref. [271] describes a DT for predicting real-time vehicle fuel consumption.

Ref. [272] introduces a holistic approach to managing logistics processes within an industrial park using a DT of the production logistics system. This study emphasizes the transportation phase within the industrial park. The proposed solution aims to synchronize information from various units in the industrial park to enable effective transportation decision-making between them. In [273], the authors suggested using digital twin in intelligent transportation systems to improve road traffic. In [274], the focus is on DTs in intelligent transport systems. Applying this technology allows for all real-world elements to be replicated in the virtual environment, including road infrastructure elements, human-driven vehicles, and autonomous vehicles. The authors of [275] have a similar goal, where the DT includes people, vehicles, and road traffic. The result of this work is a proposal for a mobility digital twin structure. In [3], a DT for managing data about road infrastructure elements is proposed. Meanwhile, ref. [276] utilized DT for rapid iteration and validation of collision avoidance systems in intelligent vehicles, enabling improved safety and reliability of such systems. Additionally, ref. [277] describes an approach to managing business processes by developing an organizational DT. This approach integrates business processes with models, ensuring reliable road freight transport in an unstable external environment. An intriguing topic is addressed in [96], which focuses on modeling driver behavior on highways using DTs as it is stated e.g., in [278]. In [98], a DT detects potential anomalies and predicts and simulates accident scenario. This topic is reviewed in [279]. A DT for forecasting passenger flows in public transport systems is presented in [280], allowing for more precise transport infrastructure planning. The authors of [281] took a comprehensive approach by proposing a telemetry platform based on a DT in the transport sector. This platform monitors fuel consumption, pollutant emissions, and driver practices.

#### 4.2.4. DTs in Supply Chain Operation

Supply chain cooperation is a crucial element for the success of many industries, both at the local and global levels. Modern challenges, such as the rapid pace of change, increasing customer demands, and the complexity of logistics operations, present new hurdles for companies. The advancement of technology and the emergence of modern solutions positively influence the visibility of the supply chain and help address these challenges. One technology that supports improving supply chain operations is the digital twin (DT). Three review papers have been published in this research area. In [282], the authors present the benefits enterprises can gain by implementing DTs in their logistics supply networks. The findings from the article demonstrated that implementing DTs would enhance visibility within their logistics supply networks. All four factors of organizational visibility (visibility for sensing, learning, coordination, and integration) would improve by developing predictive indicators, forecasts, diagnostics, and descriptions of physical resources for the enterprise’s logistics. The paper also discusses the challenges associated with DT implementation and proposes solutions to overcome them.

The second review article [283] addresses the evolution of research trends in applying DTs in supply chain management. The authors identified ten themes within these research trends. After conducting a literature analysis, it was concluded that DTs are a key factor enabling the development of resilient supply chains. Meanwhile, ref. [284] describes the impact of DTs on the supply chain and the prospects for DTs in logistics. It also presents the main barriers and opportunities for applying DTs within the supply chain. Additionally, the authors propose a framework for utilizing real-time data to generate the data streams necessary for creating a real-time operational DT.

The remaining papers selected for review in this area mainly focus on the architecture of DTs in supply chain logistics [285,286,287,288]. Furthermore, ref. [287] proposes a digital twin supply chain framework encompassing multimodal supply chains. In [289], a disruption identification model based on a DT for the supply chain is proposed. The authors of [290] developed a DT structure for risk management in logistics systems. This structure aims to create a virtual resource based on building information modeling (BIM) to monitor the ongoing progress of modular construction. The authors of [291] examined the conditions related to the design and implementation of DTs in the context of managing disruption risks in the supply chain. In [292], a DT-based intelligent cold chain management platform is described, showcasing its application in a pharmaceutical distribution center, where storage conditions, personnel safety, and product quality are monitored. Lastly, ref. [293] investigated the technical implementation of an autonomous supply chain system based on multi-agent systems (MASs) and DTs. And, at the end, Ref. [294] presents an evolutionary game model utilizing DTs for participants in a crowdsourcing logistics scenario. This learning method allows for the optimization of crowdsourced logistics.

#### 4.2.5. DTs in Water and Intermodal Transportation

In maritime transport, a key sector of the global economy, modern technologies are also being implemented. Digital twins are increasingly utilized in this sector, as they enable better management and optimization of operations, as well as improved safety and increased efficiency. A significant portion of the publications selected for this literature review focuses on monitoring the condition of technical objects.

In [295], the issue of monitoring the performance of marine engines is addressed. The authors propose a method for monitoring engine status based on a DT. After analyzing the results obtained from the engine’s DT, they confirm the validity of using this method, as the difference in accuracy between DT and real data is negligible compared to the costs incurred when monitoring engine status using other methods. Authors in [296] designed a marine engine sensor diagnostics and condition management concept. The solution proposed in the article allows for intelligent engine monitoring, advanced sensor fault detection, and precise maintenance planning. In [297], a framework for assessing the technical condition of marine engines using a DT was developed. The approach presented in [298] involves using a DT to monitor and predict fatigue damage specific to the vessel. Based on computational models that incorporate ship position data as well as meteorological and oceanographic information, it is possible to track the accumulation of fatigue in the vessel over time, make operational decisions, and plan maintenance. Additionally, in [299,300], the application of DT for diagnosing faults in autonomous water vehicles under real-world conditions is described. The case of unmanned surface vehicle DT development is given in [301]. 

In maritime transport, digital twins can also be applied at the level of port infrastructure. This enables better planning and optimization of loading and unloading processes for ships, managing traffic within the port, and forecasting resource requirements. Publications have also emerged that describe the use of DTs in intelligent ports. In [302], the authors analyze and propose directions for applying DTs in ports, focusing on their utilization in the construction and operation of ports. They also highlighted issues related to decision-making in construction within this sector. A model for managing intelligent ports based on DTs is presented in [303], where the authors explored the potential applications of DTs in managing port processes. The analysis indicates that DTs can be used to manage cargo transport operations and container terminal activities. It also facilitates risk prediction, communication, data sharing based on DTs, and managing processes to enhance environmental protection and sustainable development efforts.

A decision support system for assessing port resilience and optimizing repair activities based on a DT was introduced in [304]. Meanwhile, ref. [305] discusses optimizing lock maintenance in river systems using a DT. 

The authors in [306] presented a structure for optimizing operations and safety in transshipment terminals where a DT is employed. The application of DTs in transshipment terminal operations enhances the efficiency of processes. In another study, ref. [307] proposed the integrated maintenance decision-making model for cranes most commonly used in container terminals. A DT is utilized in this model for maintenance purposes, aiding in aligning the maintenance schedules for the analyzed equipment. In addition, ref. [308] developed a framework for monitoring the operational status of port cranes based on a DT. Lastly, ref. [309] proposed an automated structure for planning storage areas using digital twins for uncertain port shipments. This structure optimizes warehouse space, automated stacking cranes (ASCs), and automated guided vehicles (AGVs). 

In the last work [310], the authors propose and test, in a real application of an unmanned underwater vehicle (UUV), a general process for determining a subset of components needed for maintenance (triage) based on a digital twin (DT). This process leads to an increase in the reliability of the entire system. The authors frame the design problem as a multi-objective optimization problem utilizing experimentally determined data and metrics from a real UUV system model.

#### 4.2.6. DTs in In-House Logistics 

**Figure 21:**
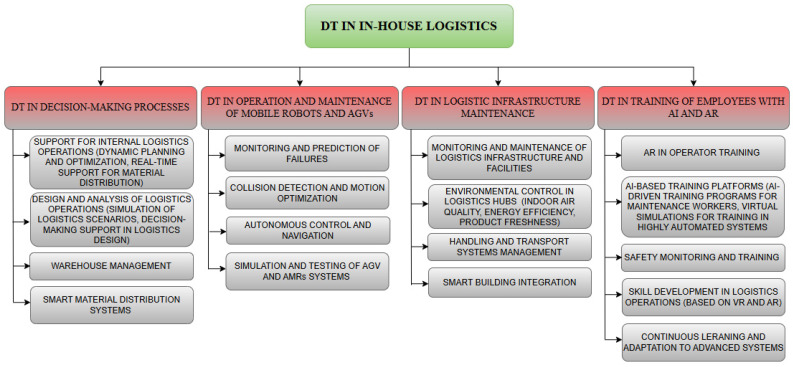
The main areas of DT implementation in in-house logistics. Source: own contribution.

Internal logistics activities are crucial to any organization, enhancing productivity and operational efficiency. At the same time, internal logistics encompasses various activities directly related to implementing material flows within the enterprise. This requires the use of various material handling and storage equipment. The classification of the main application areas of digital twins (DTs) in in-house logistics is presented in Figure 21. A review of DTs in internal transport systems is presented in Ref. [101]. Meanwhile, DTs in logistics is discussed in [99,149,311].

Authors of [312] investigate the components of a logistics hub digital twin (DT) and analyze various implementation possibilities of DTs in logistics infrastructure. In [313], network requirements for real-time data streaming and processing to generate DTs were examined. The architecture of a control system for inspection robots and methods of its implementation were also presented.

A significant portion of the topics researchers discuss regarding the use of DTs in this area focuses on mobile robots. As mentioned in [314], DTs in the maintenance of mobile robots can be used for several purposes, including predicting the battery level of a mobile robot, 3D visualization of robot calibration, and collision detection in robots. The authors of the article presented a DT that predicts when a failure will occur. In [315,316], a universal DT architecture for automated guided vehicles (AGVs) was introduced to design, manage, and forecast performance. A system for monitoring multiple mobile robots based on a DT to detect collision-prone movements in robots was presented in [317]. Meanwhile, in [318], a DT was used to propose a method for optimizing the motion trajectory of mobile robots. Control of AGVs in production systems was discussed in [319]. Additionally, an architectural structure was proposed to facilitate the automatic generation of a DT. In [320], the application of a DT for monitoring and predicting navigation errors in mobile robots was described. The DT structure presented in [145] is responsible for the remote programming and operation of AGVs. The DT model for simulating events in a system using AGVs, as explained in [321], is intended to test new vehicle management policies. In [322], the authors proposed comprehensive support for the AGV system using the DT model of that system. In the DT of the AGV system, it is possible to manage transport orders, select vehicles, and control the driving process. The focus of [323] was on evaluating the correctness of design assumptions in the early phases of deploying autonomous mobile robots (AMRs). A description of creating a DT for a control system for robots designed for inspecting complex working environments can be found in [324]. Meanwhile, ref. [325] presented a DT design for a system consisting of an assembly part and AGVs. This system allows for the selection of one of the AGVs and predicts AGV failures. A DT was also developed for robotic linear actuators to detect undesirable events [326]. In [327], a mathematical model for managing cranes’ logistics and maintenance processes was developed. In [328], a DT framework for monitoring indoor air quality was proposed. The use of a DT for scheduling and collision-free routing of AGVs in a variable environment is described in [329,330]. A model for simulating external metamorphic constraints in underground transport using mobile robots was presented in [331].

The proposal of a decision support tool based on a DT for internal logistics operations and maintenance logistics is presented in [332,333,334,335]. Authors in [336] analyzed aspects of DT application in warehouse management. In [337], the DT’s decision-making support focused on processes related to the design and analysis of logistics operations. A smart material distribution management system based on DTs was presented in [338], while logistics distribution was discussed in [339]. In article [340], the authors discuss the use of a DT for dynamic planning in large machining workshops. In [341], a DT-based monitoring and alert system for refrigerated logistics warehouses was introduced. The role of the DT, in this case, is to optimize the freshness and energy efficiency of stored products. Authors in [342] proposed a DT framework for human-building integration activities to optimize building maintenance. In [343], the design and implementation process of an evaluation algorithm for a continuous transport system DT was presented, enabling monitoring and correcting belt conveyor voltage asymmetry.

Authors of [344] introduced a remote monitoring system for augmented reality. The purpose of this system is to assist inexperienced operators in understanding data from the DT. In the context of DT application in maintenance, researchers also presented the possibility of using a DT to train new maintenance workers for highly automated systems [115]. In [345], a platform supporting a DT for monitoring safety on premises was developed.

#### 4.2.7. Other Applications

The last group of publications selected for analysis includes 15 articles that were not classified into previous groups. Some of these publications address components or parts used in various transportation sectors. On the other hand, there are also individual works related to specific industrial sectors.

The induction motor has a wide range of applications. It is used in railway, road, and internal transportation. In [346], a digital twin (DT) of the induction motor was proposed for creating databases of its failures, as diagnosing the causes of induction motor failures and monitoring its characteristics during operation is very complex. This requires collecting data from the same motor both when a failure occurs and when it does not. Meanwhile, a tool designed for the maintenance of electric motors based on a DT is presented in Ref. [347]. In [348], a DT is used for detecting inter-turn short circuits in the stator of an alternating current induction motor. Ref. [349] describes a DT for monitoring and predicting the degradation state of fuel cells. In addition, a DT structure for the reliability of lithium-ion batteries is proposed in [350].

One of the components of rotating machines, such as rotors (e.g., wheels), turbines, and internal combustion engines, is the bearing. The structure for diagnosing faults in such bearings when error data are unavailable is presented in [351]. In [352], the combination of a DT and a machine learning algorithm was developed to diagnose bearing cracks’ type and size. Similarly, in [353], the authors focused on a DT of bearings.

Several publications also address the use of DTs in maintenance within the mining industry. A DT framework for real-time monitoring of mining trucks is presented in [98]. In [354], a methodology for assessing the wear of gear tooth surfaces based on DTs is described. Ref. [355] presents the DT of the braking system in mining hoisting equipment. In [356], the possibility of using DTs to diagnose rolling bearing faults is discussed.

Article [357] describes the use of DTs in developing autonomous agricultural vehicles.

In addition, two papers focus on general complex devices. In [358], the use of a DT for assessing the technical condition of industrial machines is described. Meanwhile, the intelligent maintenance of complex devices using blockchain technology and DTs is presented in [359]. 

## 5. Discussion

The main aim of this paper is to conduct a comprehensive review of the existing literature to provide a substantive analysis within the key areas of digital twin (DT) applications in the maintenance of transport systems. A total of 201 articles meeting the established selection criteria were reviewed, allowing for an in-depth examination of the analyzed issue. Such deep analysis gives the possibility to answer the stated research questions: 

RQ1 intended to discover the leading trends in DT concept implementation in transportation systems O&P and investigate its evolution over the last decade. The main research outputs here are discussed broadly in Section 4.1 and Section 4.2.

In the defined seven application areas, the scope of issues covered is very complex, ranging from the presentation of technological solutions dedicated to predictive maintenance, condition monitoring, and forecasting to issues related to the analysis of acquired data and the need to make complex operational decisions (e.g., connected with path planning). In the studied years, the smallest number of publications regarding the use of DT technology was noted in supply chains and water and intermodal transport. These areas appear to be particularly promising for the further development of DTs. In contrast, the remaining research areas (i.e., air, rail, land, and internal transport) show a similar number of publications. Additionally, interest in these areas has been increasing over the years.

Focusing on the comparison of DT implementation across the domains of aviation, rail transport, road transport, supply chain operations, water transport, and in-house logistics reveals both similarities and differences in how this technology is leveraged to enhance operations, safety, and efficiency. The main similarities are visible in the following three main subareas: condition monitoring and predictive maintenance, process optimization, and integration with IoT and real-time data analytics. 

Across all the analyzed sectors, DTs help monitor the condition of machinery, infrastructure, and systems, enabling proactive maintenance that minimizes downtime and improves reliability. For instance, in maritime transport, a DT is used to monitor marine engines and predict fatigue damage to vessels, while in rail transport, it ensures the safety and stability of rail infrastructure. In in-house logistics, DTs monitor the status of mobile robots and equipment, ensuring smooth internal operations.

Process optimization is another key area where DTs are widely applied. Across all sectors, DT technology is leveraged to streamline operations, whether in optimizing production processes in aviation, enhancing energy management in road transport, or improving port traffic management in maritime transport. Similarly, in supply chain operations and in-house logistics, DTs are used to optimize logistics and warehouse management, leading to more efficient and effective operations.

Additionally, DTs in all these sectors are integrated with IoT systems and real-time data analytics. This integration allows for the continuous collection and analysis of data, providing valuable insights that enhance decision-making. Whether monitoring environmental conditions in logistics, predicting equipment failures in various transport modes, or managing real-time operations in supply chains, the combination of DTs with IoT and data analytics is a common and powerful tool for improving efficiency and reliability across different industries.

The main differences are summarized in Table 3.

Based on the presented summary, providing a more detailed analysis is possible. Several papers are primarily concerned with the key terms of sensors, deep machine learning, the Internet of Things, or big data analytics. In the context of implementing digital twin (DT) technology to support the management of the technical maintenance processes of transportation means, several key areas where the digital twin approach is widely applied in transportation sectors can be identified:

- Technical condition monitoring: The digital twin enables continuous monitoring of the technical condition of vehicles and transportation infrastructure. Thanks to advanced sensors and IoT technologies, the DT can collect data on part wear, engine operating parameters, and even road conditions. This allows for the quick identification of potential technical problems and failures (see, e.g., refs. [9,221]). This research problem is especially important for aircraft maintenance and risk management due to safety issues.
- Failure prediction: Based on the collected data, a DT can perform predictive analyses, forecasting future failures and technical issues. This allows for planning maintenance activities in advance, avoiding downtime and costly repairs (e.g., refs. [173,252,296]). In addition, the main leading trends here are connected with integration with advanced technologies (like BIM and IoT in railway transportation) or structural integrity maintenance. This integration enables more accurate and comprehensive monitoring of structural health by combining real-time data from DTs with the detailed digital representations offered by, e.g., BIM. The fusion of these technologies allows for early detection of structural issues, more informed decision-making, and optimized maintenance practices, ultimately enhancing the safety and longevity of infrastructure.
- Optimization of maintenance plans and schedules: Utilizing data from the digital twin, more effective maintenance plans can be developed (maintenance scheduling). A DT allows for the individual adjustment of inspection and repair schedules to the actual technical condition of vehicles, which helps reduce the maintenance costs of the transport fleet (see, e.g., refs. [307,330]). The main leading trends are connected with the integration of predictive analytics and machine learning algorithms as well as the use of IoT for continuously monitoring asset performance and allowing for dynamic adjustment of maintenance schedules.
- Simulation and testing of new solutions: A digital twin enables the simulation of various operational scenarios and the testing of new technological solutions before their implementation in real conditions. This allows for assessing potential benefits and risks associated with introducing technological innovations in the operational activities of the transport fleet (see, e.g., refs. [287,312]). One of the key challenges in the simulation and testing of new solutions is the effective integration of digital twin (DT) technology throughout the entire product lifecycle. Utilizing DTs in this context requires a seamless transition from design and development to production, operation, and eventual decommissioning. Each stage presents unique demands for data accuracy, real-time processing, and system adaptability, making it difficult to maintain a consistent and reliable digital representation of the physical product. As a result, the leading trends are connected with lifecycle integration, cross-domain collaboration, validation and verification processes, or cybersecurity. This aspect is especially important for aircraft designing and production processes.
- Optimization of fuel consumption and operational efficiency: A DT can be used to analyze and optimize fuel consumption and improve the operational efficiency of vehicles. By monitoring engine operating parameters, driver behavior, and road conditions, the DT helps identify areas needing improvement and implement effective fuel-saving strategies (e.g., refs. [292,297]). Here, one of the main trends in the implementation of the digital twin (DT) concept, particularly in optimizing fuel consumption and operational efficiency, is the increasing focus on autonomous vehicles. The integration of DT technology with autonomous systems enables real-time monitoring, simulation, and optimization of vehicle performance, leading to significant improvements in fuel efficiency. By simulating various driving scenarios and conditions, DTs help fine-tune autonomous algorithms to optimize routes, reduce idle times, and enhance overall operational efficiency. This trend is pivotal as the transportation industry moves towards greater automation and sustainability.
- Remote technical support: Using remote connections and digital interfaces, the DT allows for providing technical support by experts from anywhere in the world. This enables quick problem diagnosis and provides real-time repair instructions and guidance (see, e.g., ref. [266]). Here, one of the key challenges in implementing remote support based on digital twin (DT) technology is ensuring secure and reliable two-way communication between the physical asset and its virtual counterpart. This bidirectional communication is essential for real-time monitoring, control, and feedback mechanisms that enable effective remote support and decision-making. However, establishing and maintaining such communication channels poses significant security concerns, including the risk of data breaches, unauthorized access, and cyberattacks. To address these issues, robust encryption protocols, authentication measures, and network security strategies must be employed to protect sensitive data and ensure the integrity and confidentiality of information exchanged between the DT and the physical system. Additionally, ensuring low-latency and high-reliability connections is crucial to facilitate seamless interactions and prevent disruptions in remote support services.
- Operational data analysis of the monitored fleet: A DT allows for the analysis of data collected from the entire fleet of vehicles, which helps identify trends and patterns related to failure rates, fuel consumption, and driver behavior. This information can be used to implement improvements and optimize fleet management processes (see, e.g., ref. [184]). Leading trends in this area include the integration of big data analytics and artificial intelligence (AI) to process vast amounts of operational data in real time. This allows for more accurate predictions of fleet performance and potential issues (especially visible in railway transportation). Additionally, there is a growing focus on the use of cloud computing to store and analyze data, enabling scalable and flexible data management across entire fleets. The combination of edge computing with DTs is also becoming more prevalent, allowing for faster data processing and analysis at the source, which reduces latency and improves decision-making. Finally, the use of digital twins to create a unified data environment for the entire fleet enhances the ability to track and optimize individual vehicle performance as well as overall fleet efficiency.
- Integration with management systems: A digital twin can be integrated with existing fleet and maintenance management systems, enabling automatic data transfer and collaboration between different platforms and applications. This helps streamline operations and improve data consistency and accessibility (see, e.g., ref. [327]). Leading trends include the seamless connection of DTs with enterprise resource planning (ERP) systems and computerized maintenance management systems (CMMSs). This integration allows for real-time data exchange and better synchronization of operational and maintenance activities. There is also a trend towards incorporating DTs with predictive maintenance systems, enabling automated decision-making based on real-time data analytics. Furthermore, the use of cloud-based platforms to unify DTs with various management systems is becoming more common, facilitating centralized control and easier scalability. Another emerging trend is the integration of DTs with Internet of Things (IoT) platforms, which enhances the capability to monitor and manage assets across distributed locations.
- Safety and regulatory compliance: Implementing a DT in technical maintenance management requires addressing issues related to data security and compliance with regulatory requirements, such as data protection and occupational safety standards. Ensuring appropriate data protection measures and regulation compliance is crucial for successfully implementing the DT (e.g., refs. [280,327]). Leading trends here include using DTs to simulate and assess compliance with safety standards in real time, which helps identify potential hazards before they become critical. There is also a growing trend toward integrating DTs with automated compliance monitoring systems, enabling continuous oversight of regulatory requirements. Adopting DTs to create virtual testing environments is another trend, allowing for organizations to conduct safety drills and regulatory audits without disrupting actual operations. Additionally, DTs are increasingly being used to document and track regulatory compliance over the lifecycle of an asset, ensuring that all changes and updates are consistently managed and recorded.

The conducted systematic analysis of the selected literature makes it possible to answer the second research question.

RQ2 intended to define the main research and knowledge gaps in DT use in transportation systems operation and maintenance, especially in the context of in-house logistics. The main research outputs in this application area are discussed broadly in Section 4.2.6. Internal logistics is vital for enhancing organizational productivity and operational efficiency, involving various activities related to material flow management. Digital twin (DT) applications in this domain include mobile robots, automated guided vehicles (AGVs), and decision support tools for logistics operations. Research covers DT architectures, real-time data streaming, and predictive maintenance for mobile robots and AGVs. Additionally, DT frameworks optimize warehouse management, monitor air quality, and improve safety. Overall, the integration of DTs in internal logistics presents opportunities for innovation and efficiency gains. Indeed, in the realm of digital twin (DT) application in internal logistics, warehousing, and autonomous transportation, several knowledge and research gaps have emerged that warrant further exploration.

One prominent challenge in the field of digital twin (DT) technology is the integration of these systems with existing logistics frameworks. Although numerous studies have explored various architectural approaches for DT implementation, there is a notable absence of comprehensive frameworks that address integration challenges across diverse logistics platforms. This research gap presents an opportunity to develop best practices and guidelines for seamlessly incorporating DT technology into existing logistics systems. Research could focus on identifying and standardizing integration strategies that accommodate the variability in current logistics infrastructures, ensuring interoperability and enhancing overall system efficiency. Furthermore, the effective handling of real-time data processing within DT systems remains a significant area of concern. While some studies have addressed real-time data streaming, there is insufficient understanding of how to maintain data integrity, security, and reliability, particularly in dynamic and variable environments. This gap emphasizes the need for research into robust real-time data management methodologies. Specifically, research should explore techniques for ensuring accurate and secure data transmission, processing, and storage in scenarios involving mobile and autonomous systems where decisions are highly time-sensitive. Investigations into advanced data management solutions that can handle the complexities of real-time data in logistics and other high-stakes environments will be crucial for advancing the practical application of DT technology.

Standardization also emerges as a significant issue within the DT landscape. The development of a standardized DT framework tailored to logistics operations, including mobile robots and AGVs, is yet to be comprehensively addressed. Future research could focus on creating standardized protocols and methodologies for designing and implementing DT technology across various logistics applications, ensuring consistency and interoperability.

While the potential of DTs in predictive maintenance is recognized, gaps still exist in understanding the limitations of predictive models in varying operational contexts. Future research should focus on quantifying the reliability of predictive analytics and evaluating their performance in different logistics environments. This would help refine the application of DTs for preemptive maintenance and enhance its overall effectiveness.

Another critical area of research pertains to the scalability of DT solutions. The current literature does not adequately document how to effectively scale DT applications from small to large operations without compromising their performance. Investigating scalable models would significantly contribute to the practical implementation of DTs across diverse logistics environments. Indeed, research into scalable models is needed to facilitate the widespread adoption of DT technologies across diverse logistics settings.

User interaction and training represent notable knowledge gaps in the implementation of digital twin (DT) technologies. There is a need for a deeper understanding of how users interact with DT systems to ensure they can effectively leverage these technologies. Additionally, developing effective training programs for less experienced operators is crucial to maximize the benefits of DT systems. Future research should focus on designing intuitive user interfaces that facilitate seamless interaction with DT technologies and creating comprehensive training methodologies. These efforts will support users in making informed decisions based on DT insights, ultimately enhancing the overall utility and effectiveness of digital twin applications.

Interoperability issues between different logistics platforms and systems represent a significant challenge in the implementation of digital twin (DT) technologies. Currently, there is a notable lack of comprehensive research addressing these interoperability challenges. Research should focus on developing methods and frameworks that facilitate seamless communication and data exchange across diverse DT systems. Addressing these gaps is essential for creating a more integrated logistics ecosystem where various platforms and systems can work together efficiently. This research is crucial for enhancing logistics operations’ overall effectiveness and cohesion, ensuring that DT technologies can deliver their full potential across different environments and applications.

Furthermore, the impact of external factors, such as economic conditions, supply chain disruptions, or changes in consumer behavior, on DT performance has not been thoroughly investigated. Gaining insight into these external influences would enhance the robustness and adaptability of DT models in real-world logistics applications.

Regulatory compliance is another critical area that requires further exploration. There is currently limited research on designing digital twin (DT) applications that adhere to regulatory standards in logistics and transportation. Specifically, more studies are needed to address how DT technologies can comply with data protection regulations and operational safety requirements. Investigating these aspects is essential for promoting the responsible and effective implementation of DT technologies. Ensuring that DT systems meet regulatory standards will help safeguard data integrity, enhance safety, and facilitate broader adoption of these technologies within the logistics and transportation sectors.

Cross-disciplinary approaches integrating insights from fields such as artificial intelligence and machine learning into DT applications in logistics remain underexplored. Investigating how these technologies can enhance the capabilities of DTs could lead to innovative solutions and improved operational efficiency. There is a notable gap in understanding how AI and machine learning can be effectively combined with DT to optimize processes, predict maintenance needs, and enhance decision-making. Addressing this gap could unlock new potential for DT systems, making them more robust and versatile in handling complex logistics challenges.

Finally, the environmental impact of implementing digital twin (DT) technologies in logistics is an area that warrants further exploration. Currently, there is limited research on how DTs can be utilized to enhance sustainability practices in warehousing and transportation. This gap in knowledge highlights the need for studies that investigate how DTs can contribute to reducing environmental impacts and improving resource efficiency. Addressing these research gaps is crucial for advancing the effective implementation and optimization of DT technologies, ensuring they support sustainable practices in internal logistics, warehousing, and autonomous transportation systems. By focusing on this aspect, future research can help integrate environmental considerations into the design and deployment of DT solutions, promoting a greener logistics industry.

By addressing these knowledge and research gaps, future studies could contribute to effectively implementing and optimizing digital twin technologies in internal logistics, warehousing, and autonomous transportation systems.

RQ3 intended to discover the main aspects of DT modeling to address future challenges in the operation and maintenance of transportation systems.

Despite the evident development of modern technologies and their application in the transport industry observed over the past five years, there remains significant potential for further advancements in the area of transport maintenance. Numerous aspects can be innovated, covering both technological and organizational solutions in relation to DT concept implementation. Currently, key development directions include the following:

Predictive diagnostics: The advancement of sophisticated diagnostic systems based on artificial intelligence and data analysis enables forecasting failures in advance. This allows for planning maintenance activities before problems arise, minimizing downtime and repair costs. Main developmental trends in this area include the application of advanced machine learning algorithms. Utilizing techniques such as regression algorithms, neural networks, and decision trees allows for more accurate data analysis and identifying patterns and anomalies that may indicate potential failures. Consequently, this enhances the precision of forecasting future technical issues.

Another widely analyzed area today is the integration with vehicle monitoring systems. Predictive diagnostics can be effectively utilized in conjunction with systems that monitor the technical condition of vehicles (e.g., AGVs, mobile robots). By integrating data from various sources, such as sensors, telemetry systems, or service databases, it is possible to obtain a comprehensive picture of the technical condition of the transport fleet. Modern solutions are also moving toward ensuring two-way communication between vehicles and servers.

An essential element of predictive diagnostics is optimizing the data collection, storage, and processing processes. It is crucial to focus on key technical parameters and factors influencing vehicle reliability to obtain the most relevant information for failure forecasting. Solutions based on blockchain technology and cloud-based systems will be increasingly important in this area in the near future. Moreover, the storage of vast quantities of data poses its own challenges. Efficient and scalable data storage solutions are necessary to manage the increasing volume of data generated by predictive diagnostics systems. Traditional storage methods may not be sufficient to handle the data’s scale and complexity, necessitating the development of more advanced and adaptable storage solutions.

Automation of maintenance processes is the next research area where we may identify research gaps. Implementing robotics and automation in maintenance processes can yield numerous benefits, including improved efficiency, task execution accuracy, and elimination of human errors. Robots can be employed to perform routine maintenance tasks, allowing for staff to focus on more advanced responsibilities. The main development direction in this area is using robots, drones, and automated devices to carry out routine tasks such as mechanical inspections, cleaning, or even minor repairs.

Blockchain technology is emerging as a promising solution for ensuring data integrity and security. By providing a decentralized and tamper-proof ledger, blockchain can enhance the reliability of data used in predictive diagnostics, ensuring that it remains accurate and unaltered throughout its lifecycle.

Cloud-based systems are also becoming increasingly important in managing and processing data for predictive diagnostics. The scalability and flexibility of cloud computing enable the handling of large volumes of data and the deployment of advanced analytics tools. Additionally, cloud-based solutions can facilitate real-time data access and collaboration among stakeholders, improving the efficiency and effectiveness of predictive diagnostics.

Another trend is the implementation of advanced decision support systems based on artificial intelligence and data analysis, which allow for optimizing the planning and execution of maintenance activities. These systems can suggest optimal schedules for inspections and repairs, considering priorities, costs, and resource availability. Automating technical inspections can expedite their execution and enhance their accuracy. Employing advanced technologies like vision systems and measuring devices facilitates rapid and precise assessment of vehicle conditions, making identifying problems and planning repair actions easier. In this context, there is a search for new solutions for “smart maintenance” and proactive maintenance approaches.

Integrated inventory management: Utilizing IoT technologies and warehouse management systems allows for better monitoring and optimizing spare parts and consumables inventory levels. This helps avoid material shortages during repairs and reduces costs associated with excess inventory. The next step in building integrated inventory management systems after implementing RFID (radio-frequency identification) technology is the introduction of inventory consumption monitoring systems. RFID technologies enable precise tracking of the location and condition of spare parts in warehouses. This facilitates the quick location of needed parts and minimizes the risk of material shortages during inspections and repairs. Consumption monitoring systems allow for continuous tracking of the technical condition of parts and forecasting replacement needs, enabling preemptive maintenance actions and inventory optimization, thereby reducing fleet maintenance costs.

There is also a trend in this area toward integrating inventory management systems with diagnostic systems and implementing IoT technologies, allowing for the automatic generation of spare parts orders based on the technical condition of vehicles. This enables swift responses to alarm signals and minimizes downtime due to material shortages.

The next research area is connected with mobile technologies and remote support. The development of mobile applications and remote technical support systems allows for quick diagnosis of problems and provision of repair instructions from anywhere, increasing the efficiency of maintenance activities and reducing vehicle downtime. The foundation of today’s proactive maintenance systems is the use of mobile applications by service personnel. This grants service staff quick access to essential data, operational instructions, and repair plans. These applications can also facilitate reporting failures, logging work hours, and communicating with team members, thereby enhancing operational efficiency.

The next step involves designing and implementing remote technical support systems, enabling rapid remote diagnosis of issues and providing repair guidance from specialists regardless of location. Utilizing tools like videoconferencing and remote access to diagnostic systems allows for effective problem resolution even for vehicles located far away. Additionally, there is a growing trend towards employing augmented and virtual reality technologies to assist personnel during the execution of basic operational tasks and in training programs. This facilitates continuous skill enhancement and tailors training to individual needs and abilities.

Implementing a digital twin technology allows for simulating and monitoring vehicle behavior in real-time, leading to a better understanding of operational processes and identifying areas for improvement. In the design and implementation of fleet management systems, primary development directions will focus on developing optimization and forecasting models to minimize costs and enhance the operational efficiency of transport systems. Furthermore, literature reviews and practical implementations indicate the necessity of developing solutions that allow for inter-departmental collaboration and data integration. Implementing a digital twin requires cooperation among different departments within a company and integration of data from various information systems. With appropriate technological solutions, it is possible to obtain a comprehensive view of the technical condition of the fleet and effectively coordinate maintenance activities at all levels of the organization.

Simultaneously, fundamental innovations regarding the design and implementation of the DT concept for ensuring the reliability and maintainability of internal transport systems will encompass the following:

▪ Technological innovations—transport companies will introduce new technologies, such as AI, robotics, the Internet of Things, AR, and VR, with a DT approach to improve the efficiency and reliability of maintenance processes.
▪ Organizational innovations—aimed at introducing new management methods, work procedures, or business models (e.g., robot as a service), which enable more efficient resource utilization and enhance the effectiveness of maintenance activities.
▪ Process innovations—focused on optimizing existing maintenance processes and introducing new strategies and tools that allow for quicker responses to changes in operational conditions and minimize the risk of failures.

In conclusion, several fundamental limitations and challenges must be considered when developing and implementing the DT approach for maintaining technical systems, including the following:

▪ Technical diagnostic issues: the necessity of monitoring and collecting significant amounts of information and processing this information for proper reporting.
▪ Investment costs: Implementing modern technologies requires substantial financial investment, which will become evident through minimized repair and vehicle downtime costs due to better planning and resource utilization. The issue of investment profitability may limit companies.
▪ Data security in collection and transmission processes: ensuring the security of transmitted data and minimizing the risk of cyberattacks are key aspects to consider when implementing Industry 4.0 technologies.

The conducted systematic analysis of the selected literature makes it possible to answer the last research question.

RQ4 intended to define the framework’s scope for digital twins in the maintenance management of transportation systems. 

According to the literature review, defining a framework’s scope for digital twins (DTs) in the maintenance management of transportation systems should involve outlining objectives, key components, and functionalities that facilitate effective management and optimization of transportation assets. This review encompassed a wide range of research and case studies from different domains such as aviation, rail, road, and maritime transportation. By analyzing these diverse sources, we identified key trends, challenges, and best practices relevant to the implementation of DT technologies. This gives the possibility to define the main scope of the proposed framework. 

The need for this framework to be grounded in the existing literature while tailored for internal transportation arises from the complexity and specificity of internal logistics operations. Although the general principles and technologies discussed in the literature apply across different transportation modes, the framework adapts these principles to meet the particular needs of internal transport environments.

First, the objectives of the digital twin framework for maintenance management in transportation systems should focus on enhancing asset reliability and operational efficiency through innovative technologies. Real-time monitoring allows for continuous assessment of asset conditions, facilitating immediate detection of anomalies and issues. Predictive maintenance leverages advanced analytics to foresee potential failures, enabling proactive actions that minimize downtime and associated costs. Performance optimization enhances operational efficiencies by providing actionable insights that guide data-driven decision-making processes.

Additionally, the framework supports simulation and testing, allowing for virtual experimentation with various maintenance strategies. This capability enables organizations to evaluate the effectiveness of different approaches without disrupting actual operations, leading to improved maintenance practices.

The key components of the DT framework include the following:

1. Data acquisition and integration: This component involves collecting real-time data from various sources, including sensors, IoT devices, and existing management systems. It is crucial for creating a comprehensive digital representation of physical assets, as it enables aggregating relevant data such as operational conditions, maintenance history, and environmental factors. Effective integration of these diverse data streams ensures that the digital twin remains accurate and reflects the system’s status.
2. Data analytics and visualization: Once the data are collected, advanced analytics techniques, including machine learning and statistical analysis, are employed to derive insights. This component helps identify data patterns, trends, and anomalies, facilitating predictive maintenance and decision-making. Visualization tools play a critical role in presenting complex data in a user-friendly manner, enabling stakeholders to interpret findings and make informed decisions easily.
3. Simulation and modeling: This aspect of the framework allows for creating of virtual models that replicate the behavior of physical assets under various conditions. Through simulation, organizations can test different maintenance scenarios, evaluate the impact of potential changes, and optimize maintenance schedules. This capability not only aids in risk assessment but also supports strategic planning and resource allocation.
4. Communication and collaboration tools: Effective communication among stakeholders is essential for successfully implementing the digital twin framework. Collaborative tools enable seamless information sharing, ensuring that all team members, from maintenance personnel to management, are aligned and informed about asset status and maintenance activities.
5. Feedback mechanisms: A vital component of the digital twin framework is the establishment of feedback loops that facilitate continuous improvement. By analyzing the outcomes of maintenance actions and comparing them with the predictions made by the digital twin, organizations can refine their models and improve their predictive capabilities, leading to more effective maintenance strategies over time.

Together, these components create a robust framework that enhances the maintenance management of transportation systems, ultimately leading to increased operational efficiency, reduced costs, and improved asset longevity. In addition, these key components should be reflected in the physical and virtual layers of the DT. 

The last issue is connected with DT framework functionalities. In this area, we may distinguish six main functionalities:

▪ Condition monitoring—providing dashboards and alerts that reflect the real-time health status of assets, allowing for immediate action when anomalies are detected.
▪ Failure prediction—implementing predictive algorithms that analyze historical and real-time data to forecast potential failures and recommend maintenance actions accordingly.
▪ Maintenance scheduling—automatically generating and optimizing maintenance schedules based on predicted failure points, historical maintenance data, and operational requirements.
▪ Resource management—helping manage spare parts inventory and resource allocation by predicting the demand for parts based on the analysis of maintenance schedules.
▪ Reporting and compliance—facilitating reporting functionalities to ensure compliance with regulatory requirements and standards in maintenance practices.
▪ Feedback loop—establishing a feedback mechanism to continuously improve the digital twin models and algorithms based on actual maintenance outcomes and operational experiences.

The digital twin framework for maintenance management in transportation systems should represent a transformative approach to enhancing asset performance, optimizing maintenance strategies, and ensuring operational efficiency. As organizations increasingly adopt digital transformation strategies, integrating the digital twin framework with existing systems becomes crucial for maximizing its potential and ensuring a seamless transition.

In summary, successfully implementing the digital twin framework for maintenance management in transportation systems hinges on effective integration with existing systems and a commitment to future scalability and adaptability. Organizations can enhance their maintenance strategies and operational efficiency by creating a cohesive ecosystem that leverages historical data and encourages cross-departmental collaboration. Moreover, by designing the framework with flexibility in mind, organizations can ensure that the digital twin continues to meet their evolving needs, driving long-term asset performance and reliability improvements. This forward-thinking approach positions organizations to thrive in an increasingly complex and dynamic transportation landscape.

## 6. Framework for DTs in Transportation System Maintenance Management

The development of the framework presented in this study is a direct outcome of an extensive literature review conducted in the area of digital twin (DT) applications for transportation systems. Through a thorough examination of existing research and technological advancements, we identified key trends, challenges, and best practices relevant to the use of DTs in the operation and maintenance of transportation systems. This comprehensive review allowed for us to distill the core elements and principles necessary for creating an effective framework that addresses the common needs and objectives across different transportation domains.

The literature review highlights the growing importance of implementing digital twins (DTs) in the maintenance management of internal logistics systems, particularly internal transportation systems. In the context of effective maintenance management, DT technology plays a significant role, as it enables the evolution of maintenance strategies. Additionally, it enhances technical systems’ reliability, efficiency, and safety. Consequently, this article proposes conceptual frameworks for DTs as a tool to support key activities related to physical asset management. It presents conceptual frameworks for DTs in maintaining internal transportation systems.

**Figure 22:**
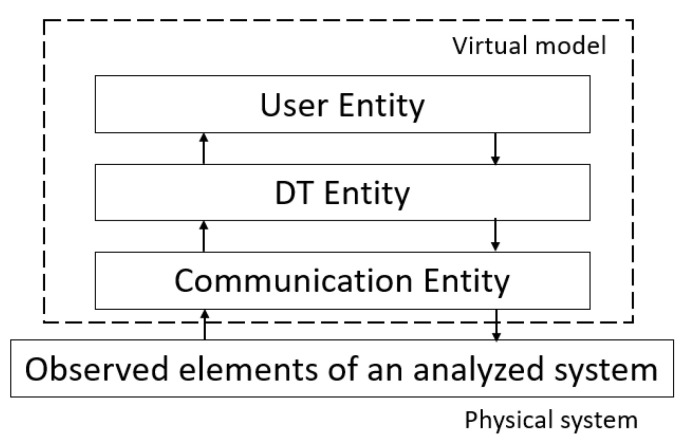
The main layers of the DT model based on ISO 23247. Source: own contribution.

In ISO 23247 [32,33,34,35], conceptual frameworks for DTs are presented, which include two interworking areas: the physical system and the virtual space. According to this standard, the conceptual frameworks consist of three main layers of the model in the virtual part and a connected layer in the real area (Figure 22).

**Figure 23:**
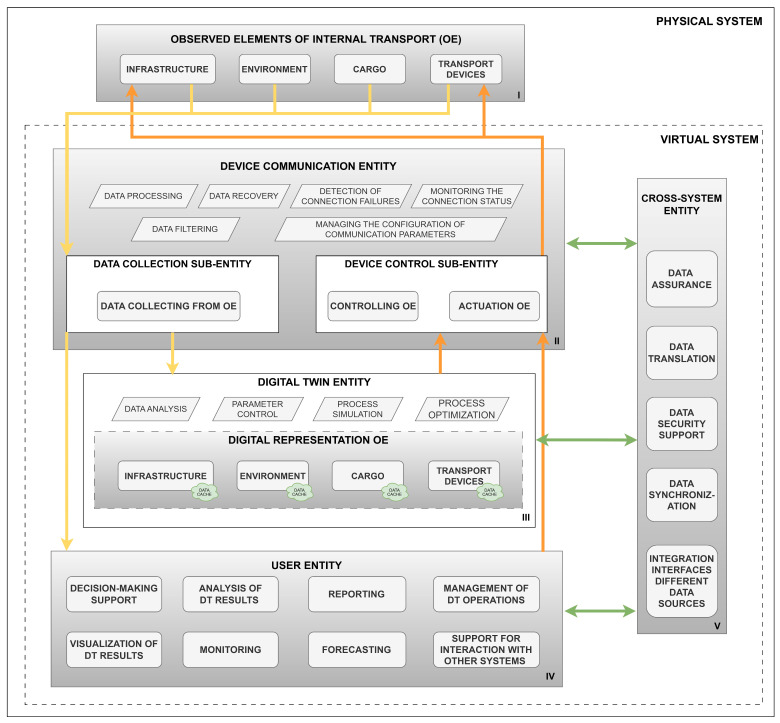
The conceptual framework for DTs in transportation systems. Source: own contribution.

Based on ISO 23247, a conceptual framework for DTs in the maintenance of internal transportation systems can be proposed (Figure 23).

The proposed conceptual framework’s first level (OE) pertains to the physical system. This includes all elements belonging to the internal transport system. Therefore, this layer encompasses not only the infrastructure of the space and its fixed elements (e.g., transport devices, storage racks) but also monitors the flow of goods and environmental conditions. These elements are continuously monitored using various measuring devices. The data collected from the OE layer form the basis for creating the virtual part of the digital twin, which is an exact replica of the real system.

Data obtained from the real system are collected and analyzed in the communication unit (Level II). This unit effectively communicates between the physical elements and their digital twin or directly with the user unit. Two subunits are distinguished in this area: the data collection subunit and the device control subunit. Data collected from the observed elements are transmitted to the digital twin unit to update the real system’s virtual copy continuously. Additionally, these data can be directly transferred to the user unit. The device control subunit controls and activates OE elements in response to requests from the user or DT units. This communication link between the real system, the user unit, and the DT unit enables the entire system to operate in two modes:

− Fully automated mode, where a closed-loop connection exists between the communication unit and the DT unit;
− Semi-automated mode, where feedback with instructions comes directly from the user unit.

The conceptual framework’s main component is the digital twin unit (Level III). Here, a virtual model of the internal transport system is developed based on data collected from Level I. This model reflects the real state and behavior of each system element. It is systematically updated based on newly collected data to ensure consistency with the actual state of the system. Additionally, this area includes a cache that stores current and historical information about each element of the real system.

The next level, Level IV—the user unit—is designed to enable employees to manage the digital twin and facilitate interpreting results generated by the DT unit. The main tasks in this area include defining maintenance goals and tasks, collecting maintenance data, and generating task commands. This unit contains functions that allow for monitoring of the OE and its digital twin and systems responsible for simulation, forecasting, data analysis, and reporting. Additionally, the user can support maintenance decision-making from this level, allowing for the system to operate in semi-automated mode. The user layer should also allow for integration with other systems and tools, enabling information exchange between different platforms.

The proposed conceptual framework also includes a cross-system entity that facilitates communication between all units in the system. Data transmitted and received must be recorded in a language understandable to the communicating units. Intermediary systems are used to translate communication protocols between different units to ensure uniform data. Additionally, integration platforms are used to facilitate data flow and state synchronization between the real system and the digital twin. This intermediary unit also includes systems responsible for supporting data security.

While the framework is grounded in the theoretical foundations and established principles from the literature, its practical implementation requires detailed consideration of specific steps and technical aspects. The framework consists of several interconnected layers and components, each playing a critical role in optimizing DT applications for internal transportation systems, and each should be carefully implemented based on the following implementation steps and technical details: 

*Physical System (Level I—OE):*

Implementation steps: Begin by identifying and cataloging all physical elements within the internal transportation system, including transport devices, storage racks, and environmental sensors. Implement continuous monitoring using advanced measuring devices to collect real-time data on asset performance and environmental conditions.

Technical details: Deploy IoT sensors and actuators for data acquisition, ensuring they are capable of interfacing with the data collection infrastructure. Integrate these data into a centralized database for further processing.

*Communication Unit (Level II):*

Implementation steps: Establish a robust data communication framework to facilitate the transfer of information between physical elements and their digital counterparts. Develop and integrate subunits for data collection and device control.

Technical Details: Utilize cloud-based systems and blockchain technology to ensure data integrity and security during transmission. Implement middleware solutions to bridge communication protocols between diverse systems.

*Digital Twin Unit (Level III):*

Implementation steps: Create a virtual model of the internal transport system using the data collected from Level I. This virtual model should be dynamically updated to reflect the real-time state and behavior of the physical system.

Technical details: Implement simulation software and data analytics tools to process and visualize the data. Ensure the model includes a data cache for storing current and historical information to support predictive maintenance and performance analysis.

*User Unit (Level IV):*

Implementation steps: Design intuitive user interfaces that allow for operators to interact with the digital twin, define maintenance tasks, and manage system operations. Provide functionalities for data analysis, simulation, and reporting.

Technical details: Develop user-friendly dashboards and decision support tools to facilitate effective management of the digital twin. Integrate the user unit with other enterprise systems to enable seamless information exchange and support semi-automated operations.

*Cross-System Integration:*

Implementation steps: develop and implement integration platforms and intermediary systems to ensure consistent communication and data exchange between different units within the system.

Technical details: Use standardized data formats and protocols to ensure uniformity across the system. Incorporate data security measures to protect against unauthorized access and ensure compliance with regulatory standards.

In summary, the framework draws from established theoretical foundations and the existing literature on DTs, ensuring it aligns with the core principles and trends identified in scholarly research. For instance, it incorporates the DT concept as defined by ISO 23247 [32,33,34,35], which outlines a structured approach with interworking areas including the physical system and the virtual space. This adherence to theoretical standards ensures that the framework is grounded in established research while being adaptable to practical applications in internal transportation systems.

In addition to theoretical integration, the framework is designed to complement asset management concepts. It incorporates essential elements of asset management, such as the continuous monitoring and evaluation of physical assets, the use of data for predictive maintenance, and the optimization of maintenance strategies. By doing so, it aligns with the broader asset management framework, enhancing the reliability, efficiency, and safety of internal transportation systems. This alignment underscores the framework’s role in advancing asset management practices through the application of DT technologies.

The development of this framework also directly addresses several identified research gaps in the literature. For example, current literature indicates a lack of standardized protocols for implementing DTs across various domains. Our framework proposes standardized approaches and methodologies for designing and implementing DT systems, ensuring consistency and interoperability within internal transportation systems.

In addition, the challenge of scaling DT solutions from small-scale implementations to large operations is a significant gap. Our framework addresses this by providing scalable models that ensure performance is maintained across diverse operational scales.

Understanding user interactions with DT systems and designing effective training programs are crucial for maximizing technology benefits. The framework includes provisions for user-friendly interfaces and comprehensive training methodologies to support effective decision-making.

The framework tackles interoperability issues by creating methods for seamless communication and data exchange between different DT systems, fostering a more integrated logistics ecosystem.

By addressing these gaps, the framework not only builds upon the insights and standards established in the literature but also advances the practical application of DT technologies in internal transportation systems. It serves as a comprehensive tool that bridges theoretical principles with practical asset management needs, thereby contributing to the effective implementation and optimization of digital twin technologies in diverse logistical contexts.

## 7. Conclusions

This article presents a systematic literature review addressing the main areas of digital twin utilization in the operation and maintenance of transportation systems. The analysis of 201 recent publications from 2012 to 2024, along with a review of publication trends, allowed for a discussion of specific applications of DT in the transportation sector.

The presented work suffers several limitations, mostly related to the reviewing methodology assumptions connected with publication collection, searching strategy, and filtering criteria. Here, the most notable limitation is associated with the used keywords as a search engine. Despite using a broad spectrum of keywords, some works connected with transportation sector O&M processes may be omitted. Indeed, despite efforts to cover a broad range of terminology related to DTs, variations in terminology across different fields or research groups may lead to gaps in capturing all relevant aspects of DTs. In addition, the literature related to medicine, health issues, or environmental aspects is omitted in the conducted overview analysis. The authors focused on logistics, transportation, and supply chain-related publications. Moreover, the conducted literature analysis does not consider the quality of the investigated publications based on times cited. The authors present only the most cited keywords in their bibliometric analyses. Another limitation may be connected with the geographical scope of the reviewed studies. A majority of the sources come from China, which may potentially restrict the global applicability of the results. What should also be underlined is that the authors focused on publications like peer-reviewed articles and conference papers, possibly overlooking significant research found in industry reports, white papers, or theses that might provide additional insights into digital twin (DT) applications. The last aspect is connected with the comparability of the selected publications. The diversity in methodologies across the reviewed studies could impact the comparability of results and affect the general conclusions drawn about the effectiveness and application of DTs. 

The systematic literature review identified seven fundamental research areas within the transportation sector where the use of DTs for maintenance has been analyzed. The conducted literature review highlighted several key findings. One of the most important is that modern service and maintenance methods, along with the role of digital twins, are key factors in ensuring transportation fleets’ durability, maintainability, and reliability. Adapting to the changing technological landscape and fostering collaboration among various stakeholders in the industry are essential for further improving fleet service and maintenance processes. Continued discussion and cooperation can contribute to introducing innovative solutions to ensure a safe and efficient transportation infrastructure for future generations.

The authors’ future research direction may involve addressing the challenges that could arise when implementing a digital twin framework in internal transportation systems within real enterprises. Additionally, future steps should include exploring potential issues related to integrating such frameworks, such as interoperability, data security, and the need for standardization across different systems.

The authors recommend a more exhaustive literature review for future work, especially related to domain-specific areas. Furthermore, topics such as sustainable maintenance systems or mitigating environmental impacts related to new technology implementation may require further exploration. Another interesting research direction may be connected with developing a new business model. In this area, concepts such as “Machine as a Service” or “Software as a Service” are other trends suitable for future research.

## Figures and Tables

**Table 3:** A summary of the main differences identified in the analyzed transportation sectors in the context of DT implementation.

Transportation Sector	Scope and Complexity of Application	Specific Use Cases	Technological Integration and Challenges
**Aviation**	DT applications in aviation are broad and complex, encompassing design, production, fleet management, and training. The use of advanced technologies like VR/AR and complex data analytics is prominent in this sector.	DT applications range from predicting component life to modeling risks in critical areas such as fatigue.	Integrates advanced technologies like VR, AR, and complex algorithms to handle extensive data and simulations.
**Rail transport**	The focus is more on infrastructure monitoring (e.g., tracks, switches) and vehicle safety, emphasizing maintaining the rail network’s stability and reliability.	DT technology is heavily used for infrastructure and vehicle monitoring, focusing on safety and maintenance optimization.	Emphasizes reliable and stable infrastructure, integrating a DT with predictive maintenance and safety systems.
**Road transport**	DTs in road transport, particularly with electric and autonomous vehicles, focus on simulating vehicle operations, optimizing energy use, and enhancing safety through predictive analytics.	DT applications are significant in electric vehicles and autonomous systems, particularly in battery management and route optimization.	Involves integrating DTs with AI and machine learning for vehicle autonomy and energy management.
**Supply chain operations**	DTs are critical for managing logistics, risk, and ensuring visibility across the entire network, often integrating with multi-agent systems and predictive technologies.	DT technology aids in logistics management, risk assessment, and the integration of real-time data to improve the resilience and efficiency of supply chains.	Focuses on integrating DTs with logistics systems for real-time visibility and risk management, often dealing with the complexity of multi-agent environments.
**Water transport**	DTs are used at both the vessel level (e.g., engine monitoring, fatigue prediction) and the port infrastructure level, optimizing port operations and traffic management.	DT applications are seen in both vessel management and port infrastructure, focusing on operational efficiency, safety, and sustainability.	Incorporates DTs at both macro (port management) and micro (vessel maintenance) levels, dealing with maritime-specific challenges like environmental impact and operational safety.
**In-house logistics**	This sector uses DTs to optimize internal processes, particularly in managing mobile robots (AGVs), warehouse operations, and indoor environmental conditions.	The focus is on optimizing internal transport systems, robot management, and environmental control, with DTs providing crucial support for operational efficiency and maintenance.	The integration of DTs with robotics and automated systems is crucial, with a focus on enhancing productivity and safety within controlled environments.

## Data Availability

Not applicable.

## Supplementary Materials

The following supporting information can be downloaded at: https://www.mdpi.com/article/10.3390/s24186069/s1, Table S1: The PRISMA checklist.

## Abbreviations

The following abbreviations are used in this manuscript:

**Abbreviation**	**Full Description**
AGVs	Automated guided vehicles
AI	Artificial intelligence
ASCs	Automated stacking cranes
BIM	Building information modeling
DEVOTION	Developing digital twin systems with automated model management
DM	Digital model
DRL	Deep reinforcement learning
DS	Digital shadow
DT	Digital twin
DTI	Digital twin instance
DTP	Digital twin prototype
EDT	Experimentable digital twin
ESDT	Early-stage digital twin
FMEA	Failure mode and effect analysis
IoT	Internet of Things
MAS	Multi-agent system
ML	Machine learning
OE	Observed elements of internal transport
O&M	Operation and maintenance
PRISMA	Preferred Reporting Items for Systematic Reviews and Meta-Analyzes
RIS	Reconfigurable intelligent surface
SLR	Systematic literature review
TDT	Transportation digital twin
UAV	Unmanned aerial vehicle
UUV	Unmanned underwater vehicle
VR	Virtual reality
